# Genome-Wide Association Study and Genomic Prediction for Soybean Cyst Nematode Resistance in USDA Common Bean (*Phaseolus vulgaris*) Core Collection

**DOI:** 10.3389/fpls.2021.624156

**Published:** 2021-06-07

**Authors:** Ainong Shi, Paul Gepts, Qijian Song, Haizheng Xiong, Thomas E. Michaels, Senyu Chen

**Affiliations:** ^1^Department of Horticulture, PTSC316, University of Arkansas, Fayetteville, AR, United States; ^2^Department of Plant Sciences, University of California, Davis, Davis, CA, United States; ^3^United States Department of Agriculture, Agricultural Research Service, Beltsville Agricultural Research Center, Beltsville, MD, United States; ^4^Department of Horticultural Science, University of Minnesota, St. Paul, MN, United States; ^5^Southern Research and Outreach Center, University of Minnesota, Waseca, MN, United States

**Keywords:** common bean, *Phaseolus vulgaris*, soybean cyst nematode, *Heterodera glycines*, genomic prediction, genome wide association study, genomic selection, single nucleotide polymorphism

## Abstract

Soybean cyst nematode (SCN, *Heterodera glycines*) has become the major yield-limiting biological factor in soybean production. Common bean is also a good host of SCN, and its production is challenged by this emerging pest in many regions such as the upper Midwest USA. The use of host genetic resistance has been the most effective and environmentally friendly method to manage SCN. The objectives of this study were to evaluate the SCN resistance in the USDA common bean core collection and conduct a genome-wide association study (GWAS) of single nucleotide polymorphism (SNP) markers with SCN resistance. A total of 315 accessions of the USDA common bean core collection were evaluated for resistance to SCN HG Type 0 (race 6). The common bean core set was genotyped with the BARCBean6K_3 Infinium BeadChips, consisting of 4,654 SNPs. Results showed that 15 accessions were resistant to SCN with a Female Index (FI) at 4.8 to 9.4, and 62 accessions were moderately resistant (10 < FI < 30) to HG Type 0. The association study showed that 11 SNP markers, located on chromosomes Pv04, 07, 09, and 11, were strongly associated with resistance to HG Type 0. GWAS was also conducted for resistance to HG Type 2.5.7 and HG Type 1.2.3.5.6.7 based on the public dataset (*N* = 276), consisting of a diverse set of common bean accessions genotyped with the BARCBean6K_3 chip. Six SNPs associated with HG Type 2.5.7 resistance on Pv 01, 02, 03, and 07, and 12 SNPs with HG Type 1.2.3.5.6.7 resistance on Pv 01, 03, 06, 07, 09, 10, and 11 were detected. The accuracy of genomic prediction (GP) was 0.36 to 0.49 for resistance to the three SCN HG types, indicating that genomic selection (GS) of SCN resistance is feasible. This study provides basic information for developing SCN-resistant common bean cultivars, using the USDA core germ plasm accessions. The SNP markers can be used in molecular breeding in common beans through marker-assisted selection (MAS) and GS.

## Introduction

Common bean (*Phaseolus vulgaris* L.) is the most important edible grain legume crop worldwide, with crop value equal to the combined value of all other food legumes such as peas and chickpeas (Jain et al., 2016). The most common bean is harvested as seed grain called “dry bean,” but it is also grown as a green vegetable (called “green bean” or “snap bean”) in many parts of the world. Common bean has high nutritional value and is one of the most important sources of protein for billions of people in the world. In recent years, about 2 million acres were planted, and approximately 1.3 million metric tons of common beans valued at US$2 billion were produced annually in the United States (US) (USDA NASS, 2020).

The production of dry beans in the US may be challenged by an emerging, invasive pest, the soybean cyst nematode (SCN), *Heterodera glycines* Ichinohe (*Tylenchida: Heteroderidae*). The SCN is the most serious pathogen of soybean [*Glycine max* (L.) Merr.] in the US and suppresses a yield more than any other pathogen (Koenning and Wrather, 2010; Allen et al., 2017). The SCN reduces a yield by feeding on plant nutrients, retarding root growth, reducing water and nutrient uptake and transportation from roots to shoots, and inhibiting rhizobium nodulation. Yield losses can exceed 40% (Koenning and Wrather, 2010), depending on many factors such as SCN population density, soil texture and fertility, rainfall, and the presence of susceptible soybean genotypes (Duan et al., 2009). The SCN has been widely spread in the US, especially in the North Central region that produces most soybeans (Tylka and Marett, 2017). Unfortunately, the top four common bean-growing states, North Dakota, Michigan, Nebraska, and Minnesota, which produce approximately 70% of the common bean in the US, are also in the North Central region. The SCN has been reported in the common bean fields of those states (Poromarto et al., 2010; Yan et al., 2017). SCN infection can cause severe yield loss without any aboveground symptoms in common beans (Poromarto et al., 2010, 2012) and becomes a serious threat to common bean production.

The use of host resistance has been highly successful in SCN management for soybeans. Numerous commercial SCN-resistant soybean cultivars are available and are planted in most soybean fields in the US. Similarly, the use of host resistance in common bean cultivars will also be crucial to SCN management in dry bean production. Growing common bean cultivars resistant to SCN infection will not only reduce common bean yield loss but also relieve SCN pressure for soybean production if common beans and soybean are rotated with wheat (*Triticum aestivum* L.). Recently, Osorno et al. (2020) has released the first pinto bean cultivar “ND Falcon,” a new pinto bean with combined resistance to SCN and rust. Screening more common bean germplasm for SCN resistance, using different HG Types (races) will provide breeders to use germplasm as parents to develop and release new superior common bean cultivars with broad and more stable resistance.

Limited research has demonstrated that some common bean germplasm and cultivars are resistant to SCN. Smith and Young (2003) conducted a greenhouse study to evaluate 20 common bean lines for SCN resistance and found a few lines resistant to SCN, and some Mesoamerican genotypes were more resistant than Andean genotypes. Poromarto et al. (2012), in North Dakota, evaluated 416 accessions (germplasm lines) in the USDA core collection of *P. vulgaris* and found 23% of the lines had low nematode reproduction and were considered highly resistant to SCN HG Type 0 (Jain et al., 2016, 2019). Wen et al. (2019), in Illinois, evaluated 363 accessions of the same core collection and found 19 accessions (5%) were highly resistant to SCN HG Type 2.5.7, and 160 (44%) resistant to HG Type 1.2.3.5.6.7, with FI < 10.

Jain et al. (2016) analyzed the transcriptome sequences of the SCN-resistant line PI533561 vs. SCN-susceptible *P. vulgaris* line GTS-900 and demonstrated that genes-encoding nucleotide-binding site leucine-rich repeat resistance (NLR) proteins, WRKY transcription factors, pathogenesis-related (PR) proteins, and heat shock proteins involved in diverse biological processes were differentially expressed between SCN-resistant and susceptible genotypes. Recently, two reports on SCN-resistant quantitative trait loci (QTLs) in common beans were published. Wen et al. (2019) conducted a genome-wide association study (GWAS) based on the dataset of 363 USDA common bean core accessions phenotyped against SCN HG types 2.5.7 and 1.2.3.5.6.7 and genotyped, using 84,416 single nucleotide polymorphisms (SNPs) obtained from genotyping by sequencing (GBS) and reported that there were five SNPs on chromosome Pv01 and one on Pv09 associated with resistance to HG Type 2.5.7. They also reported a gene cluster orthologous to the three genes at the SCN-resistant *rhg1* locus in soybeans. In addition, an SNP was found on Pv09, associated with resistance to HG Type 1.2.3.5.6.7. Jain et al. (2019) conducted GWAS in 317 accessions of USDA common bean core collection, challenged with SCN HG Type 0, and found 14 significant SNP markers on Pv04, 05, 06, 07, 08, 10, and 11 in the Middle American subpopulation and 23 SNP markers on Pv01, 02, 07, 08, 09, and 11 for the Andean subpopulation. Besides, Jain et al. (2019) reported several candidate genes on Pv01 and Pv08, which had high similarity to the three genes of *rhg1* of soybean for SCN resistance. Based on previous reports and the study, the SCN resistance in the common bean is polygenic traits with multiple genes or alleles.

Plant molecular breeding has been the foundation for crop improvement into the twenty first century and has become part of the breeding programs to expedite advances and genetic gains in many crops (Moose and Mumm, 2008). Marker-assisted selection (MAS) has been successfully used in the selection of specific major genes/alleles in plant breeding (Collard et al., 2005; Collard and Mackill, 2008; Xu and Crouch, 2008). More recently, predictive breeding *via* GS has become an essential tool in crop improvement. GS refers to selecting the performance of individuals within a population based on genomic-estimated breeding values (GEBV) (Hayes et al., 2009; Desta and Ortiz, 2014). The decreasing cost of DNA sequencing renders GS affordable and powerful by providing high-density markers across the genome (Lin et al., 2014). GS is more efficient than the traditional MAS when dealing with small-effect QTL (Bernardo and Yu, 2007; Heffner et al., 2009, 2011; Cortés et al., 2020). So far, genomic prediction (GP) as a GS parameter has been investigated in a dozen of crops such as maize (*Zea mays* L.), rice (*Oryza sativa* L.), soybean, and wheat (Bernardo and Yu, 2007; Heffner et al., 2009, 2011; Albrecht et al., 2011; Jarquin et al., 2014, 2016; Onogi et al., 2016; Xavier et al., 2016; Shikha et al., 2017; Zhang et al., 2017; Qin et al., 2019) for various agronomic traits, and abiotic and biotic stress traits. Genomic breeding value estimation in GP is the key step in GS. Several approaches have been proposed for GEBV, such as BLUP methods (gBLUP, RR-BLUP, cBLUP, and sBLUP) and Bayesian methods (BayesA and BayesB). All articles discussed the selection prediction accuracy (PA), estimated using the Pearson's correlation coefficient (r) between the GEBV and observed values for each trait in validation sets (testing sets), using several models. In recent years, GP has also been reported in common beans to predict agronomic traits under different environmental stresses (Keller et al., 2020) and SCN resistance (Wen et al., 2019).

Currently, SNP technology is the molecular-marker platform of choice in genome-wide mapping, association studies, diversity analysis, and tagging of important genes in plant genomics and breeding. SNPs are abundant in the genome, cost-effective, and amenable to high throughput analysis (Collard and Mackill, 2008; Xu and Crouch, 2008). Therefore, the identification of SNP markers will provide breeders with powerful tools to assist in selecting biotic and abiotic stress resistance/tolerance and expedite the development of elite cultivars with stress resistance/tolerance in common bean breeding programs. SNPs have been reported and used in common beans (Cortés et al., 2011; Blair et al., 2013). Gene-based SNP markers were developed in common beans (Galeano et al., 2012). SNP genetic maps for common beans have been constructed, using the 6K SNP BeadChips (Song et al., 2015) and were used to anchor the scaffold of the common bean whole-genome sequence reference assembly for the Andean landrace G19833 (Schmutz et al., 2014). In common beans, the BARCBean6K_3 Infinium BeadChip has been used for QTL and association mapping to identify genes/QTL controlling different traits (Hagerty et al., 2015, 2016; Hoyos-Villegas et al., 2016, 2017; Moghaddam et al., 2016; Castro et al., 2017; Hurtado-Gonzales et al., 2017; Valentini et al., 2017). Recently, several versions of *P. vulgaris* (common bean) genome assembles were released. They include the aforementioned Andean genome (Schmutz et al., 2014; https://phytozome-next.jgi.doe.gov/info/Pvulgaris_v2_1) and four Middle American genomes: (1) race Mesoamerica: cultivar OAC Rex (https://www.ncbi.nlm.nih.gov/genome/380?genome_assembly_id=1500596) and breeding line BAT93 (https://www.ncbi.nlm.nih.gov/genome/380?genome_assembly_id=262776; Vlasova et al., 2016; Rendón-Anaya et al., 2017); (2) race Durango: cultivar Pinto UI111 (https://phytozome-next.jgi.doe.gov/info/PvulgarisUI111_v1_1), and cultivar Labor Ovalle of race Guatemala (https://phytozome-next.jgi.doe.gov/info/PvulgarisLaborOvalle_v1_1). The genome assembly of G19833 has been used as a reference to map SNPs of the BARCBean6K_3 Infinium BeadChip to the 11 chromosomes in common beans (Song et al., 2015).

With the decreased genotyping cost and improved statistical methods, GWAS and GS offer new approaches for genetic improvement of complex traits in crop species. GWAS, based on a population of unrelated lines and high-density markers, has been used to identify candidate genes for a broad range of complex traits in different crops (Huang et al., 2010; Li et al., 2013; Morris et al., 2013; Yano et al., 2016). GWAS is relatively new for common beans, but it has been reported to be an effective approach to identify SNP markers associated with SCN resistance (Jain et al., 2019; Wen et al., 2019). However, MAS has been successfully coupled with backcrossing schemes for transferring several traits, among which anthracnose resistance and seed mineral accumulation traits (even from the wild) in common beans (Garzón et al., 2008; Blair and Izquierdo, 2012). Therefore, research is needed to identify SNP markers associated with SCN resistance and to use these SNP markers in molecular breeding to enhance common bean improvement.

We initiated a project in 2016 to study the SCN resistance in common beans, using SCN HG Type 0. So far, two studies for SCN resistance QTLs in the USDA common bean core collection have been reported (Jain et al., 2019; Wen et al., 2019). Wen et al. (2019) conducted GWAS in 363 accessions of USDA common bean core collection phenotyped against SCN HG Types 2.5.7 and 1.2.3.5.6.7 and genotyped, using GBS. The common bean core sets were also genotyped BARCBean6K_3 Infinium BeadChip SNPs, and the SNP data are available (Song et al., 2015; Gepts et al., 2019; Kuzay et al., 2020). The BARCBean6K_3 Infinium BeadChip could provide additional SNPs for a breeding program. Therefore, we conduct GWAS and GP analysis for resistance to HG Type 2.5.7 and HG Type 1.2.3.5.6.7, using the phenotypic data from Wen et al. (2019) and genotypic data of the BARCBean6K chip SNPs in this report. Although Jain et al. (2019) conducted GWAS in 317 accessions of USDA common bean core collection with SCN HG Type 0, only 86 accessions with FI < 10 were published in the article; hence, their data are not included in this study for further analysis. The overall goal of the research was to develop technology to effectively manage the SCN in common bean productions. Specifically, the objectives of this study were to evaluate the SCN resistance in the USDA common bean core collection, to conduct GWAS, and to identify SNP markers associated with SCN resistance. The approach was to first conduct GWAS to identify associated SNP markers and then use the associated SNP markers to do GS. This is an approach combining MAS and GS through GEBVs, using associated SNP markers (Spindel et al., 2016; Zhang J. P. et al., 2016; Qin et al., 2019; Ravelombola et al., 2019, 2020, 2021; Ali et al., 2020). The information presented in this report is a new contribution to the understanding of SCN resistance in common beans beyond the previous studies (Jain et al., 2019; Wen et al., 2019).

## Materials and Methods

### Plant Materials

About 315 common bean germplasm accessions, a core set of common beans, described at USDA Germplasm Resources Information Network (GRIN), were used in this study. This common bean core set has been widely used for genetic diversity analysis (Kwak and Gepts, 2009; McClean et al., 2012; Campa et al., 2018; Gepts et al., 2019; Kuzay et al., 2020). The core set was mainly from two gene pools, i.e., the Andean and Mesoamerican pools (Gepts et al., 1986, 2019; Koenig and Gepts, 1989; Koinange and Gepts, 1992; Beebe et al., 1997, 2000; Kwak and Gepts, 2009; Bitocchi et al., 2012, 2013; McClean et al., 2012; Schmutz et al., 2014; Campa et al., 2018; Kuzay et al., 2020), and can form three clusters and seven groups (Kuzay et al., 2020). The 315 accessions in this study were originally collected from 11 countries, including Mexico (163 accessions), Colombia (35), Guatemala (30), Peru (17), Costa Rica (17), Ecuador (16), El Salvador (13), Nicaragua (13), Honduras (9), Bolivia (1), and United States (1) (Supplementary Table 1) They represented 241 accessions from Middle American gene pools, 67 from the Andean pool, and seven from an admixture (Supplementary Table 1).

In addition, the seven soybean SCN HG Type indicator (differential) lines PI 548402 (Peking), PI 88788, PI 90763, PI 437654, PI 209332, PI 89772, and PI 548316 (Niblack et al., 2002), and four SCN race differential lines PI 548402 (Peking), PI 548982 (Pickett 71), or PI 548988 (Pickett), PI 88788, and PI 90763 (Riggs and Schmitt, 1988), with the susceptible Williams 82 (PI 518671) as control were included to confirm the virulence phenotype of the SCN population (Supplementary Table 2). Based on the reactions of the differential lines to the SCN population, the population was HG Type 0 and race 6 similar to race 3.

### Soybean Cyst Nematode Resistance Phenotyping

The 315 common bean accessions were tested for their resistance to SCN HG Type 0 (race 6). HG Type 0 is avirulent to most current commercial SCN-resistant soybean cultivars, and if there is any SCN resistance in common beans, it is likely resistant to HG Type 0 based on the knowledge of SCN resistance in soybeans. Consequently, we started screening, using the HG Type 0, to identify more SCN-resistant common bean lines and genes/alleles.

The SCN population was collected from a field in Swift County, Minnesota, USA, in 2007. Since it was collected from the field, the population had been maintained in the greenhouse on susceptible soybean cultivars or stored in a freezer at −20°C. Prior to the experiment, the nematode population was cultured on susceptible soybean “sturdy” for about 45 days. Newly formed females and cysts were washed with a vigorously applied water stream through an 850-μm-aperture sieve onto a 250-μm-aperture sieve and extracted by centrifugation in a 63% (w/v) sucrose solution. Eggs were released from the cysts by crushing the cysts on a 150-μm-aperture sieve with a rubber stopper mounted on a motor (Faghihi and Ferris, 2000). The eggs were separated from debris by centrifugation in a 35% (w/v) sucrose solution for 5 min at 1,500 g, and an egg suspension of 800 eggs/ml was made. The reproduction of the SCN population on the soybean or bean lines was assayed by growing the bean in cone-tainers (4-cm diameter and 13.5-cm high) in a growth room (Supplementary Figure 1).

The experimental design was a randomized complete block design (RCBD) with three replicates. Each replicate included two common bean plants in two separate cone-tainers per common bean accession. Control soybean Williams 82 in each replicate included five plants in five separate cone-tainers. All three replicates of the 315 common bean accessions, with a total of 1,890 cone-tainers, plus the Williams 82, were set up within 2 days of December 14 to 15, 2016, in the growth room, and they were arranged in three blocks (Supplementary Figure 1). The cone-tainers were filled with autoclaved soil (80% sands + 20% field clay loam soil) to half to which 2,000 eggs in 2.5 ml of water were added. Additional soil was placed in the cone-tainer to approximately 2 cm from the top. Another inoculum of 2,000 eggs in 2.5 ml of water was added to the soil surface, and one common bean or soybean seed was sowed in each cone-tainer. The seed was covered with additional soil to about 1-cm depth. The cone-tainers were placed on a rack and maintained in the growth room with the temperature set at 28°C and daily artificial lights for 16 h. Water was applied with a sprinkler irrigation system to maintain adequate soil moisture (Supplementary Figure 1). The environments, including soil temperature, moisture, and lights, were controlled relatively even over time and across the benches in the growth room. After 35 days in the growth room, the plants were cut to about 1 cm above the soil surface, and all of the cone-tainers were moved to a cool room (4°C) to stop SCN development. The samples were stored in the cool room until processed.

Cysts (females) were extracted from the roots and soil according to established procedures after 35 days. Briefly, the soil and plant roots were removed from the cone-tainer and placed in a beaker, and water was added. Any cysts on the wall of the cone-tainer were washed off. Plant roots were removed and females washed off on an 850-μm-aperture sieve, nested on a 250-μm-aperture sieve. In addition, the cysts in the soil were extracted by pouring soil suspension on the sieves. After rinsing the materials on the 850-μm-aperture sieve, the cysts with debris on the 250-μm-aperture sieve were collected. The cysts were separated from the debris by flotation centrifugation in sucrose solution (63%) and counted under a dissecting microscope.

A FI for each common bean plant was determined by comparing SCN female number of the plant with the average female number on the five plants of Williams 82: FI = female number on a given plant × 100/mean number of females on Williams 82, where we defined FI on Williams 82 as 100. The average FI of the two plants in each block was considered as one replicate, and three replicates were included.

So far, two studies for SCN resistance in the USDA common bean core collection have been reported (Jain et al., 2019; Wen et al., 2019). Wen et al. (2019) conducted GWAS in 363 accessions of USDA common bean core collection phenotyped against SCN HG Types 2.5.7 and 1.2.3.5.6.7. Among the 363 accessions reported in Wen et al. (2019), 276 accessions were further analyzed for GWAS and GP in this report based on available SNP data. Therefore, we also include their SCN FI data in this study for comparative data analysis. Although Jain et al. (2019) conducted GWAS in 317 accessions of USDA common bean core collection with SCN HG Type 0, only 86 accessions with FI < 10 were published in the article; hence, their data are not included in this study for further analysis.

### Phenotypic Data Analysis

The SCN resistance phenotypic data FI of SCN HG Type 0 (race 6) among the 315 common bean accessions were analyzed, using the ANOVA, with the general linear models (GLM) procedure of JMP Genomics 7 (SAS Institute, Cary, NC). For comparisons among individual accessions in JMP, the “LSMeans Student's *t*” was used to perform multiple comparisons at α = 0.05. The mean, range, SD, SE, and coefficient of variation (CV) were estimated for FI, using “Tabulate.” Person's correlation coefficients (r) were calculated, using “Multivariate Methods.” The distribution of FI was drawn, using “Distribution” in JMP Genomics 7. The average of FI to SCN HG Type 0 (race 6) for each soybean accession from ANOVA was used as the phenotypic data for GWAS.

The broad-sense heritability (H) was estimated, using the following formula (Holland, 2003).

$$\begin{array}{l} \text{H}=\sigma^{2}\text{g}/\left[\sigma^{2}\text{g}+\;\left(\sigma^{2}\text{e}/\text{r}\right)\right] \end{array}$$

with $\sigma_{\text{g}}^{2}$ being the total genetic variance, $\sigma_{\text{e}}^{2}$ being the residual variance, and r being the number of blocks. The estimates for $\sigma_{\text{g}}^{2}$ and $\sigma_{\text{e}}^{2}$ were [EMS(G)–Var (Residual)]/r and Var (Residual), respectively. EMS(G) and Var (Residual) were obtained from the ANOVA table.

### Genotyping

The common bean core set was genotyped with the BARCBean6K_3 Infinium BeadChips (Song et al., 2015), consisting of 5,398 SNPs distributed across the 11 pairs of common bean chromosomes with the Illumina BeadStation 500G (Gepts et al., 2019; Kuzay et al., 2020). The 5,389 SNPs across 382 accessions of the common bean core set are available and can be downloaded on the website at https://datadryad.org/stash/dataset/10.25338/B8KP45, with AA BB AB—format. The AA BB AB—was changed to the nucleotide format (A C T G) based on *P. vulgaris* G19833 reference sequences. After elimination of the missing data, a total of 4,654 SNPs were used for genetic diversity, population structure analysis, and GWAS in this study with a missing rate <20%, heterogeneous <10%, and minor allele frequency (MAF) > 5%. The distribution of the 4,654 SNPs on the 11 chromosomes of the common bean is shown in Supplementary Figure 2.

### Genetic Diversity and Population Structure Analysis

This collection was previously analyzed with simple-sequence repeats (SSRs) (McClean et al., 2012) and SNPs (Gepts et al., 2019; Kuzay et al., 2020) for their genetic diversity and population structure. They found mainly three or seven subpopulations in the core set. In this study, we repeat the genetic diversity and population structure in the 315 accessions from the core set. A model-based clustering method in the STRUCTURE 2.3.4 program (Pritchard et al., 2000) was used to infer the population structure of the common bean accessions based on the 4,654 SNPs. To identify the number of populations (K) capturing the major structure in the data, the burn-in period was set at 50,000, with the Markov Chain Monte Carlo iterations, and the run length was set at 10,000 in an admixture model; correlated allele frequencies were assumed to be independent for each run (Lv et al., 2012). Ten runs were performed for each simulated value of K, ranging from 1 to 10. For each simulated K, the statistical value delta K was calculated, using the formula described by Evanno et al. (2005). The optimal K was determined, using Structure Harvester (Earl and Vonholdt, 2012; http://taylor0.biology.ucla.edu/structureHarvester/). Each common bean genotype was then assigned to a cluster (Q) based on the probability determined by the software that the genotype belonged in the cluster. The cutoff probability for assignment to a cluster was 0.50 or above. Based on the optimum K, a bar plot with “Sort by Q” was obtained to show the population structure among the common bean genotypes (accessions).

The number of principal components (PC) was chosen according to the optimum subpopulation determined in STRUCTURE 2.3.4, and a PCA plot was drawn, using R package ggplot2 by the data from TASSEL 5 (Bradbury et al., 2007; http://www.maizegenetics.net/tassel). Genetic diversity also was assessed, and phylogenetic trees were drawn, using MEGA 7 (Kumar et al., 2016) based on the Maximum Likelihood (ML) tree method with the following parameters (Shi et al., 2016, 2017): the bootstrap method with the number of bootstrap replications 500; model/method: the General Time Reversible model; rates among sites: Gamma distributed with Invariant sites (G + I); the number of discrete gamma categories: five; gaps/missing data treatment: Use all sites; the ML heuristic method: Subtree-Pruning-Regrafting-Extensive (SPR level 5); the initial tree for ML: Make the initial tree automatically (Neighbor-Joining); and a branch swap filter: Moderate. During the drawing of the phylogeny trees, the population structure and the cluster information were imported to MEGA 7 for combined analysis of genetic diversity. For the sub-tree of each Q (cluster), the shapes of “Node/Subtree Marker” and the “Branch Line” were drawn with the same color as in the figure of the bar plot of the population clusters from the STRUCTURE 2.3.4 analysis.

### Association Analysis

GWAS was performed, using the Genomic Association and Prediction Integrated Tool version 3 (GAPIT3) (Lipka et al., 2012; Wang and Zhang, 2020; https://zzlab.net/GAPIT/index.html; https://github.com/jiabowang/GAPIT3), where the mixed linear model (MLM), compressed MLM (CMLM) (Zhang et al., 2010), GLM, SUPER (Wang et al., 2014), multiple-locus MLM (MLMM), Fixed and Random Model Circulating Probability Unification (FarmCPU) (Liu et al., 2016), and Bayesian-information and Linkage-disequilibrium Iteratively Nested Keyway (BLINK) (Huang et al., 2019) were run in this study. Single marker regression (SMR), GLM (Q), and MLM (Q+K) were also conducted, using TASSEL 5 (Bradbury et al., 2007; http://www.maizegenetics.net/tassel). Q-matrix (Q) was obtained from the population structure analysis by STRUCTURE 2.3.4, and Kinship (K) was estimated by the tool Kinship with the Scald_IBS method built-in TASSEL 5. In addition, a *t*-test was performed for every single SNP, using visual basic codes in Microsoft Excel 2016. Multiple modes in several tools were used to identify SNP markers associated with resistance to SCN HG Types to recognize more sTable NP markers and to tag the candidate gene(s) or QTL region(s) strongly associated with the SCN resistance. Highly significant associations were determined, using a strict Bonferroni correction of *P*-value at an α = 0.05, in which the *P* = 0.05/ (SNP number) as the significance threshold (López-Hernández and Cortés, 2019). In this study, for the panel of all 315 accessions, the significant LOD [−log_10_ (*P*-value)] [LOD was used instead of −log_10_ (*P*-value) in the text] threshold value was 4.97, 4.84, and 4.52 for the panel of all 315 accessions, Q1, and Q2, respectively, based on the 4,654 SNPs, 3,455 SNPs, and 1,653 SNPs used for each panel after filtered with a missing rate <20%, heterogeneity <10%, and MAF > 5%.

Besides the SCN phenotypic data of resistance to HG Type 0 (race 6) in the USDA common bean core collection from the experiment used to conduct GWAS for SCN resistance, the phenotypic data of resistance to HG Types 2.5.7 and 1.2.3.5.6.7 from the report by Wen et al. (2019) were also used to conduct GWAS, using the same BARCBean6K_3 Infinium BeadChips (Song et al., 2015). Although Wen et al. (2019) conducted the GWAS for the two HG Types, using 84,416 SNPs identified from GBS, more information and more SNP markers would be provided that are associated with resistance to HG Types 2.5.7 and 1.2.3.5.6.7 when using different SNP sets and different GWAS models. An LD heat map was drawn for regions containing a significant SNP marker, using Haploview (Barrett et al., 2005; https://www.broadinstitute.org/haploview/haploview). However, we do not conduct an LD-based haplotype association analysis in this research.

### Candidate Gene Prediction

Candidate gene models were searched within 50 kb on either side of significant SNPs (Zhang H. Y. et al., 2016) and retrieved from the reference annotation of the common bean genome reference Pvulgaris v1.0_218 (https://genome.jgi.doe.gov/portal/pages/dynamicOrganismDownload.jsf?organism=Phytozome) because the SNP information was based on this reference sequence (Gepts et al., 2019).

### Genomic Prediction of SCN Resistance

In this study, the ridge regression best linear unbiased prediction (RR-BLUP) was used to predict GEBV in GP and performed in the rrBLUP package (Endelman, 2011), with the R software Version 4 (https://cran.r-project.org/bin/windows/base/rtest.html). The RR-BLUP is an effective and accurate prediction method as demonstrated in a wide range of traits and crops (Jarquin et al., 2014; Zhang J. P. et al., 2016). In additions, GP was performed with the genomic best linear unbiased prediction (gBLUP) (Wang and Zhang, 2020; https://zzlab.net/GAPIT/index.html; https://github.com/jiabowang/GAPIT3) and also performed using Bayesian models: Bayes A, Bayes B, Bayes LASSO (BL), and Bayes ridge regression (BRR) (Legarra et al., 2011; Barili et al., 2018), random forest (RF) (Ogutu et al., 2011), and support vector machines (SVM) (Maenhout et al., 2007). The “Bayesian Generalized Linear Regression (BGLR),” “RF,” and “kernlab” were used and run in the R package to perform the GP models for Bayes A, Bayes B, BL, BRR, RF, and SVM (Bao et al., 2014; Ravelombola et al., 2019, 2020, 2021).

In this study, we conducted four groups of GP analyses (Bao et al., 2014; Tan et al., 2017; Ravelombola et al., 2019, 2020, 2021). (1) GP was performed with six different ratios of a training set: a testing set with 19:1, 9:1, 4:1, 2:1, and 1:1; or as 5, 10, 20, 30, 40, and 50% of a testing set in the panel of 315 common accessions. Each training population subset was randomly selected from the association panel, and the remainder was used as a testing set. (2) Nine different SNP number sets from 20 SNPs to all 4,654 SNPs were used in cross-predictions of resistance to three HG Types, using five GP models: rrBLUP, Bayes A, Bayes B, BL, and BRR. (3) Six different testing set sizes (percentages) from 5 to 50% were used in cross-prediction for resistance to three HG Types in three common bean populations (all tested accessions, Q1 population, and Q2 population), using a rrBLUP model. (4) Three SNP sets (all 4,654 SNPs, 20 SNP markers, and 20 random SNPs) were used in cross-prediction of resistance to three HG Types, using eight GP models (rrBLUP, gBLUP, Bayes A, Bayes B, BL, BRR, RF, and SVM). The PA was estimated using the average Pearson's correlation coefficient (r) between the GEBVs and observed phenotypic values for SCN resistance in the validation set (testing set) (Zhang J. P. et al., 2016; Shikha et al., 2017). The r-value indicates PA and the selection efficiency of GP; the higher the r-value, the more PA and the better the selection efficiency in GS. The training and testing sets were randomly created 100 times, and the r-value was estimated each time. The average r-value of 100 times was calculated for each trait (here for SCN HG Type 0, 2.5.7, or 1.2.3.5.6.7). The distribution charts were drawn by Microsoft Excel 2016 and R package ggplot2.

## Results

### Soybean Cyst Nematode Resistance Evaluation

The reactions of SCN HG Type indicator lines and race differential lines to the SCN population are presented in Supplementary Table 2. In the HG Type test, the susceptible control Williams 82 soybean yielded 289 averaged SCN females per plant, indicating there was adequate SCN reproduction for this study. All of the seven HG Type indicators were resistant with FI < 10, confirming that the SCN used in this study was the HG Type 0 (Supplementary Table 2). In the race test, the susceptible control Williams 82 soybean yielded 426 averaged females per plant, indicating there was adequate reproduction for this study. The lines PI 548982 (Pickett 71) and PI 548988 (Pickett) were moderately resistant to the SCN population with FI 19.3 and 25.6, respectively; and other indicator lines were resistant with FI < 10, confirming the population was race 6 (Supplementary Table 2).

**Figure 1:**
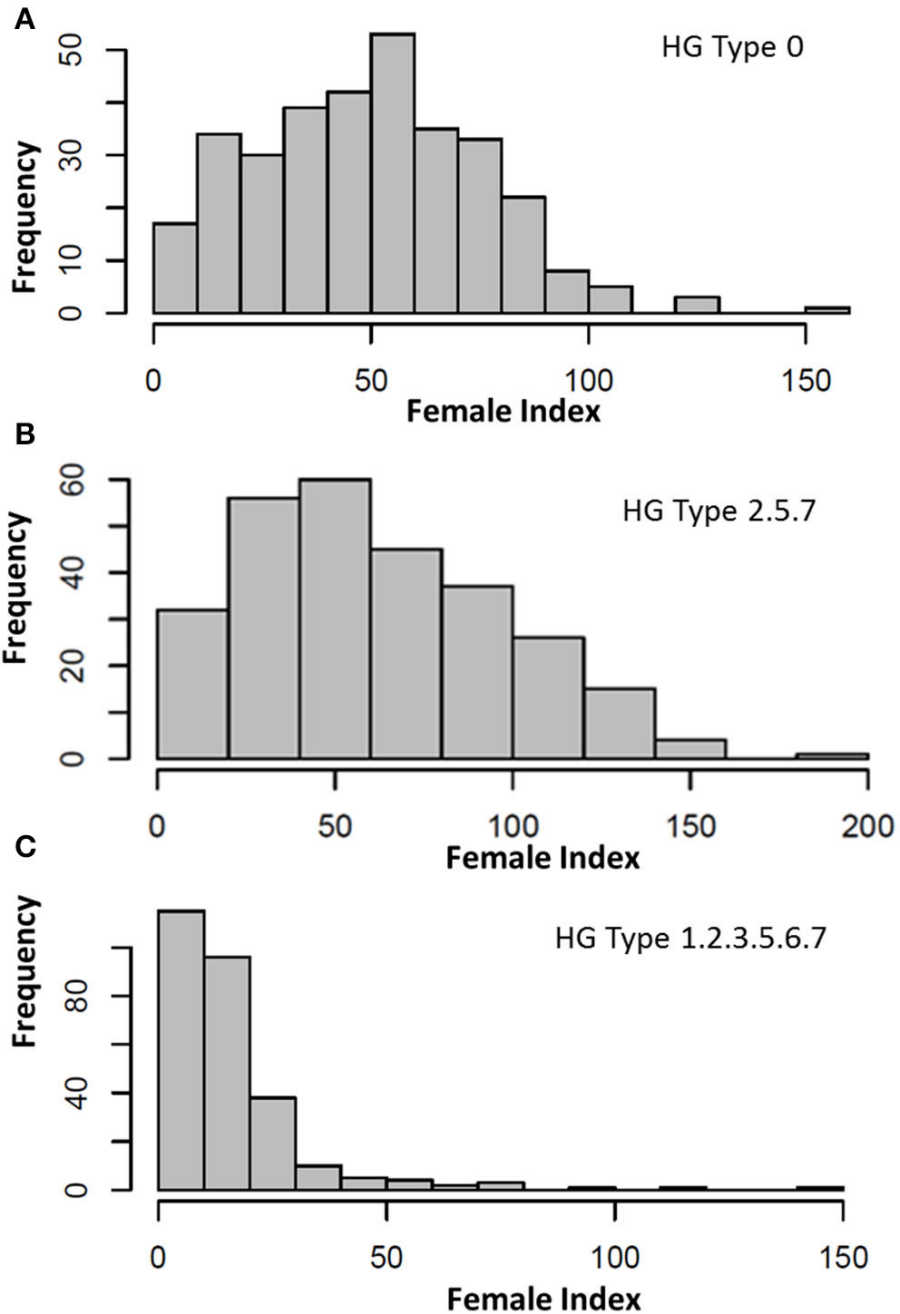
Distribution of female index (FI) of soybean cyst nematode (SCN) HG Type 0 (race 6) **(A)**, HG Type 2.5.7 **(B)**, and HG Type 1.2.3.5.6.7 **(C)** on 315 USDA common bean germplasm core collection.

The FI of the HG Type 0 (race 6) on the common bean core accessions had a large range (145.5) from 4.8 on PI 313733 to 150.3 on PI 313671 (Supplementary Tables 1, 3, Figure 1), with an average of 49.9, SD of 25.45, SE of 1.43, and CV of 51.0%; and a near-normal distribution (Figure 1A), indicating a large variation of resistance reactions to the SCN HG Type 0. Fifteen accessions were resistant to the HG Type 0 with FI < 10. The top seven accessions with the highest resistance to HG Type 0 were PI313733, PI201329, PI319684, PI313440, PI325614, PI417616, and PI313445, with FI ranging from 4.8 to 6.7, and the two most susceptible accessions were PI313671 with FI 150.3 and PI182004 with FI 124.5 (Supplementary Tables 1, 3). The H was 65.7%, indicating the HG Type 0 resistance was highly inheritable.

The FI of HG Type 2.5.7 ranged (199.1) from 0.4 on PI 313445 to 199.6 on PI 313671 (Supplementary Tables 1, 3, Figure 1), with an average of 62.9, SD of 36.4, SE of 2.19, and CV of 50.1%; a skewed near-normal distribution (Figure 1, middle) indicated a large variation of resistance reactions to this SCN HG Type. Twelve accessions were resistant to the HG Type 2.5.7 with FI < 10. The top seven accessions with the highest resistance were PI313445, PI417754, PI430210, PI201354, PI415913, PI417616, and PI325653, with FI ranged from 0.4 to 4.0; the two most susceptible accessions were PI313671, with FI 199.6 and PI 307820, with FI 158.6 (Supplementary Tables 1, 3).

The FI of HG Type 1.2.3.5.6.7 had a large range (146.1) from 0 for five accessions to 146.2 for PI 207148 (Supplementary Tables 1, 3, Figure 1), with an average of 15.9; SD of 17.0; a skewed distribution (Figure 1, bottom) indicated there was a large variation of resistance reactions to this SCN HG Type. Fifty-nine out of the 276 accessions (21.4%) had FI < 5.0, and 115 out of 276 accessions (41.7%) had FI < 10, indicating there was a high percentage for the accessions resistant to the HG Type 1.2.3.5.6.7 (Supplementary Table 1). Many accessions were classified as resistant or highly resistant to HG Type 1.2.3.5.6.7, and only eight were susceptible (FI > 65). The two highest susceptible entries were PI207148 with FI 111.4 and PI313671 with FI 146.2.

Combining analysis of resistance to the three HG Types, only one accession, PI 313671, was susceptible with high FI > 100 for the three HG Types, indicating this accession can serve as a susceptible control. Four accessions, namely, PI201354, PI 313445, PI417616, and PI313733, had FI < 10 for resistance to the three HG Types, suggesting they have high and broad resistance to the three HG Types 0, 2.5.7, and 1.2.3.5.6.7 (Supplementary Table 1). There were 37 accessions with resistance to the three HG Types (FI < 20: Table 1); their genetic diversity will be analyzed in the following section of this report.

**Table 1:** Accession ID, origin (country), population clusters and groups, and their SCN Female Index (FI) of top 37 SCN-resistant common bean accessions in reaction to HG Types 0, 2.5.7 and 1.2.3.5.6.7.

**Line_ID^a^**	**Line**	**Country**	**2Q_cluster**	**2_group**	**HG_Type 0_FI**	**HG_Type 2.5.7_FI**	**HG_Type 1.2.3.5.6.7_FI**
PI313615b_Colombia_Q1_0.987_0.013^a^	PI313615	Colombia	Q1	I	14.80		
PI313630b_Colombia_Q1_1_0	PI313630	Colombia	Q1	I	9.15		
PI309845_Costa Rica_Q1_1_0	PI309845	Costa Rica	Q1	I	11.66	28.19	4.49
PI343950_Guatemala_Q1_1_0	PI343950	Guatemala	Q1	I	8.10		
PI449410_Mexico_Q1_1_0	PI449410	Mexico	Q1	I	14.54		
PI313328b_Mexico_Q1_1_0	PI313328	Mexico	Q1	I	7.02		
PI201329_Mexico_Q1_1_0	PI201329	Mexico	Q1	I	5.06	10.57	2.24
PI201354_Mexico_Q1_1_0	PI201354	Mexico	Q1	I	7.19	3.08	0.37
PI417667_Mexico_Q1_1_0	PI417667	Mexico	Q1	I	11.71	24.23	16.82
PI313440_Mexico_Q1_1_0	PI313440	Mexico	Q1	I	5.92	8.81	17.2
PI313445_Mexico_Q1_1_0	PI313445	Mexico	Q1	I	6.74	0.44	0.1
PI313444_Mexico_Q1_1_0	PI313444	Mexico	Q1	I	7.08	16.74	10.28
PI325630_Mexico_Q1_1_0	PI325630	Mexico	Q1	I	15.73	9.25	3.36
PI417616_Mexico_Q1_1_0	PI417616	Mexico	Q1	I	6.46	3.96	7.29
PI313473_Mexico_Q1_1_0	PI313473	Mexico	Q1	I	10.38		
PI203920_Mexico_Q1_1_0	PI203920	Mexico	Q1	I	19.41	25.55	12.71
PI313501_Mexico_Q1_1_0	PI313501	Mexico	Q1	I	8.33	23.79	0.56
PI325642_Mexico_Q1_1_0	PI325642	Mexico	Q1	I	11.22	10.13	3.74
PI313512_Mexico_Q1_1_0	PI313512	Mexico	Q1	I	12.95	14.1	7.48
PI201296_Mexico_Q1_1_0	PI201296	Mexico	Q1	I	14.11	12.78	1.87
PI313490_Mexico_Q1_1_0	PI313490	Mexico	Q1	I	19.25	27.75	2.24
PI325653_Mexico_Q1_1_0	PI325653	Mexico	Q1	I	16.21	3.96	1.5
PI417739_Mexico_Q1_1_0	PI417739	Mexico	Q1	I	19.76	13.22	1.12
PI430206_Mexico_Q1_1_0	PI430206	Mexico	Q1	I	9.40	12.33	0.1
PI313820_Mexico_Q1_0.989_0.011	PI313820	Mexico	Q1	I	11.08		
PI313425_Mexico_Q1_1_0	PI313425	Mexico	Q1	I	15.09		
PI417657_Mexico_Q1_0.89_0.11	PI417657	Mexico		I	14.56	22.03	3.93
PI430204_Mexico_Q1_0.692_0.308	PI430204	Mexico		II(I)	13.89	14.1	4.49
PI346960_Mexico_Q1_0.661_0.339	PI346960	Mexico	Q1(2)	II(I)	14.30	12.33	12.9
PI345576_Costa Rica_Q1_0.672_0.328	PI345576	Costa Rica	Q1(2)	II(I)	11.06	15.86	0.56
PI241794_Ecuador_Q2_0.119_0.881	PI241794	Ecuador	Q2	II	14.55	20.7	15.89
PI415936_Ecuador_Q2_0.027_0.973	PI415936	Ecuador	Q2	II	10.73	13.66	12.34
PI209498_Costa Rica_Q2_0.019_0.981	PI209498	Costa Rica	Q2	II	11.47	28.19	17.01
PI313733_Mexico_Q2_0_1	PI313733	Mexico	Q2	II	4.78	5.73	4.49
PI325731_Mexico_Q2_0_1	PI325731	Mexico	Q2	II	17.58		
PI316030b_Peru_Q2_0_1	PI316030	Peru	Q2	II	13.51		
PI293355_Peru_Q2_0_1	PI293355	Peru	Q2	II	18.04	27.31	10.09

a *Line_ID consists of PI accession, original country, one of the two clusters Q1 or Q2, the Q1 probability, and Q2 probability. For example, PI313615b_Colombia_Q1_0.987_0.013, where the PI accession is PI313615b, which is grouped into Q1 cluster with probabilty of 0.987 and has 0.013 probability to Q2*.

There were weak correlations (r = 0.31 to 0.33) of SCN resistance to HG Types, 0, 2.5.7, and 1.2.3.5.6.7 resistance among the 315 common bean accessions (Supplementary Table 4), suggesting that they had different genetic resistance to the three HG types.

From the 86 common bean accessions reported by Jain et al. (2019), 59 accessions were also screened for their resistance to HG Type 0 in this study. Six out of the 59 lines, PI201354, PI201329, PI430206, PI319684, PI343950, and PI269209, showed HG Type 0 resistance with FI < 10 in both studies, indicating the six lines had more durable or stable resistance. However, the correlation of the SCN HG Type resistance in the 59 lines between the two studies was very low, with r = 0.057, indicating that the SCN pathogens used in the two studies might have different pathogenicity. It is possible that the HG Type 0 population used in Jain et al. (2019) and the population we used belonged to different races because HG Type 0 can be race 3 or 6, and the race of the former was not reported.

### Genetic Diversity and Population Structure Analysis

The population structure of the 315 USDA germplasm accessions was initially inferred, using STRUCTURE 2.3.4 (Pritchard et al., 2000). The peak delta K was observed at K = 2, indicating the presence of two main population clusters, Q1 and Q2, in the common bean germplasm panel (Supplementary Figures 3A,B). The classification of accessions into populations or clusters based on the model-based structure from STRUCTURE 2.3.4 is shown in Supplementary Figure 3B and Supplementary Table 1. The 315 accessions were assigned to one of the two populations or clusters, defined as Q1 and Q2 groups (populations). Q1 and Q2 consisted of 248 (78.7%) and 67 (21.3%) accessions, respectively (Supplementary Table 1). Seven accessions were classified as Q1(2) because their probability belonging to Q1 was >0.5 but <0.7 (Supplementary Table 1, bottom). A graphical plot of the PCA of the 315 common bean accessions showed two clusters (Supplementary Figure 3C) based on data from TASSEL 5 with two subpopulations.

The genetic diversity among the 315 accessions was also assessed, using the ML method in MEGA 7 (Kumar et al., 2016), with phylogenetic trees are drawn based on the results. All accessions were assigned into one of the two clusters (populations), further indicating there were two distinct genetic populations in the common bean core set.

**Figure 2:**
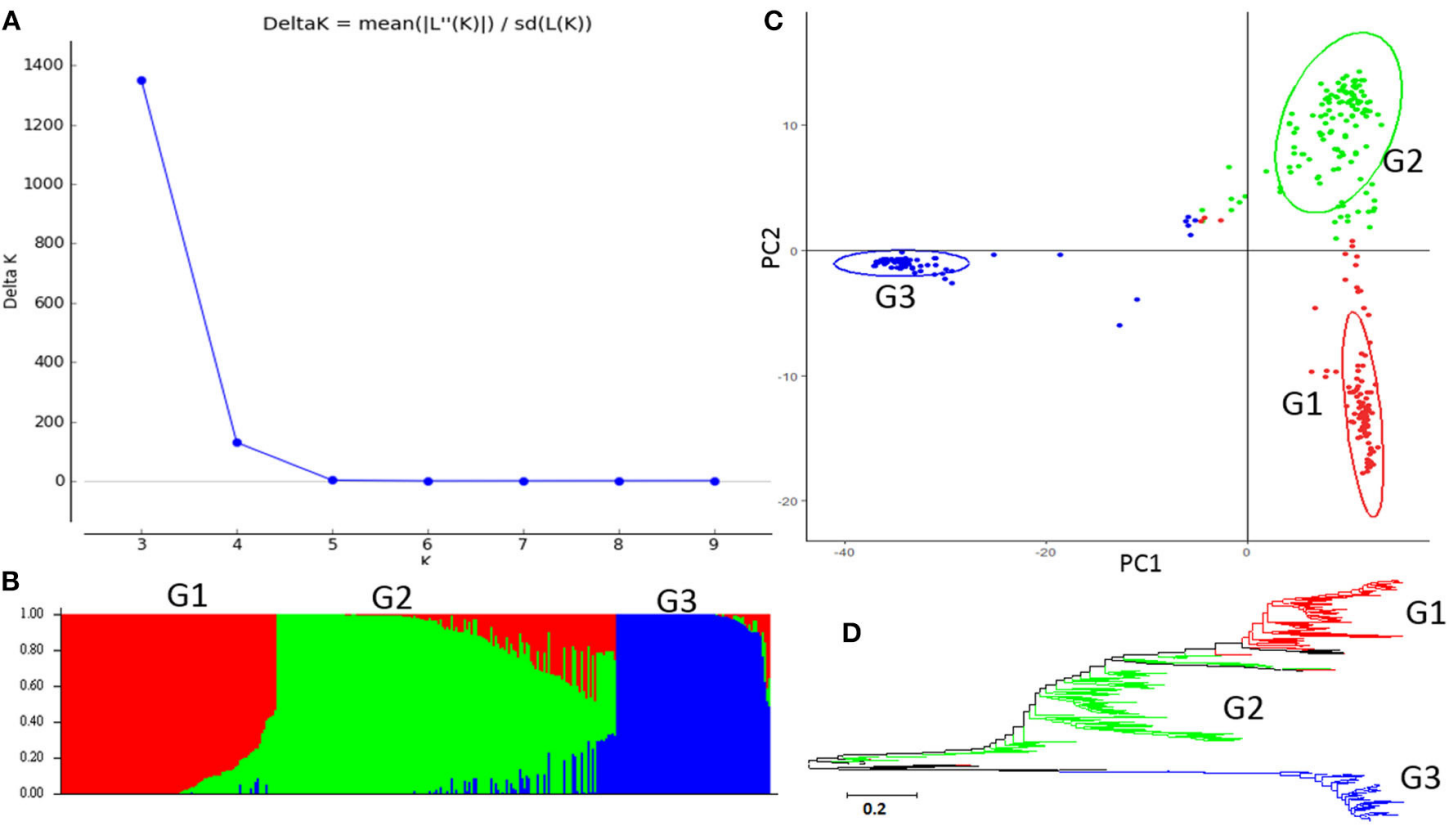
Model-based populations in the association panel consisted of 315 USDA common bean germplasm accessions. **(A)** Delta K values for different numbers of populations (K) assumed in the analysis completed with the STRUCTURE Version 2.3.4 software. **(B)** Classification of 315 common bean accessions into three populations using the STRUCTURE Version 2.3.4, where the numbers on the y-axis show the subgroup membership and the x-axis shows the different accessions. The distribution of accessions in different populations is indicated by the color coding (Cluster 1, G1, is red; Cluster 2, G2, is green; and Cluster 3, G3, is blue). **(C)** Graphical plot of the principal component analysis (PCA) of the 315 common bean accessions. The horizontal and vertical axes are the first and second principal components, and the variances explained by each component are noted. **(D)** Maximum Likelihood (ML) tree of the 315 common bean accessions drawn in MEGA 7. The color code for each population is consistent in the **(B–D)**.

The second highest peak of delta K in STRUCTURE 2.3.4 was observed for K = 3, using Structure Harvester, indicating the 315 common bean germplasm accessions can be divided into three clusters (G1 to G3) (Figure 2A). Figure 2B shows the bar plot drawn in STRUCTURE 2.3.4 to visualize the three-clustered populations. The classification of the germplasm accessions into populations based on the model-based structure developed in STRUCTURE 2.3.4 was shown in Figure 2B, Supplementary Table 1. Each common bean accession also was assigned to one of the three populations based on probabilities calculated in STRUCTURE 2.3.4 (Supplementary Table 1). A Q value = 0.5 was used to divide the three populations (clusters) and the admixture. In total, 301 out of 315 accessions (95.6%) were assigned to one of the three populations. G1 to G3 consisted of 97 (30.8%), 138 (43.8%), and 66 (21.0%) accessions, respectively. The remaining 14 accessions (4.4%) were categorized as having admixed ancestry between G1 and G3 (Supplementary Table 1). A PCA plot was shown in Figure 2C based on data from TASSEL 5.

The genetic diversity of the 315 common bean accessions was also analyzed, using the ML method in MEGA 7 by combining the three populations G1 to G3, identified by STRUCTURE. The results shown in Figure 2D indicate there may be three differentiated genetic populations and admixtures among the 315 accessions.

Combining (1) the two subpopulations (Q1 and Q2) and (2) the three clusters (G1 to G3) from STRUCTURE 2.3.4, a rectangular phylogenetic tree was drawn, using the ML method from MEGA 7 (Supplementary Figure 4). The common bean accession number, the original country of the accession, and the two populations (clusters) were merged into one taxon name for each branch in the combined tree drawn by MEGA 7 (Supplementary Figure 4). The resulting tree shows there were three main groups: (1) Q1G1, (2) Q1G2, and (3) Q2G3 in the 315 accessions (Supplementary Figure 4). Q1G1 included 96 accessions (30.5%), Q1G2 138 accessions (43.8%), Q1G (admixture) 8 (2.5%), Q2G3 66 (21.0%), Q2G31 (admixture) 1 (0.3%), and Q1(2) Gx (admixture) 7 (2.2%), indicating that the Q1 population was further divided into two groups and some admixture. The entire Q2 group (except one) was not subdivided into the K = 3 analysis and became group G2 (G2~ = Q2~ = Q2G2), with only one exception (Supplementary Table 1), suggesting the Q2 population has a well-defined genetic background with stable boundaries.

### Association Analysis

In this study, we performed GLM, MLM, SUPER, MLMM, FarmCPU, and BLINK analyses in GAPIT3 by setting PCA = 3, and SMR, GLM (Q), and MLM (Q+K) analyses in TASSEL 5, where Q = 3. We also conducted a *t*-test for each SNP. If an SNP had a LOD [−log (*P*-value)] greater than the significance threshold value LOD [−log (0.05/SNP number)] in one of the six MLM models (gapit.mlm, gapit.mlmm, gapit.super, gapit.farmCPU, gapit.blink, or tassel.mlm), the SNP was selected as a candidate-associated SNP marker and listed in Supplementary Tables 5–7 for resistance to SCN HG Types 0, 2.5.7, and 1.2.3.5.6.7, respectively. After combining the output from GAPIT3 and Tassel 5 for the three association panels (all tested accessions (all.set), Q1 and Q2 populations), the SNP markers, which were significant for resistance to the three HG Types, are listed in Table 2.

**Table 2:** SNP markers associated with three SCN HG Types, 0, 2.5.7, and 1.2.3.5.6.7 in three sets of common bean genotypes, based on six models, BLINK, FarmCPU, MLM, MLMM, SUPER, and GLM in GAPIT 3 and three models, MLM, GLM, and SMR in Tassel 5, and *T*-test.

**SNP**	**Chr**	**Position**	**–log(*****P*****-value) using GAPIT 3**						**–Log(P-value) in Tassel**			***T*-test**	**Rsq in Tassel**			**R-allele**	**S-allele**	**MAF (%)**	**Set**	**Associated_HG_Type**
			**Blink**	**FarmCPU**	**MLM**	**MLMM**	**SUPER**	**GLM**	**SMR**	**GLM**	**MLM**	**–LOG(P)**	**SMR**	**GLM**	**MLM**					
ss715640464	4	33307678	10.31	4.31	4.90	1.08	0.79	6.27	0.03	5.74	4.16	0.38	0.05	7.47	6.33	T	C	22.6	all.315	HG Type 0
ss715650114	6	10550456	5.68	5.11	2.60	2.70	2.36	2.91	3.04	2.32	1.97	3.38	4.39	3.09	2.94	T	G	26.6		
ss715647158	7	7343812	6.78	3.31	4.24	3.31	1.19	5.07	7.15	5.56	4.40	5.72	10.01	7.20	6.70	C	A	8.7		
ss715649511	7	7759866	5.76	3.36	2.43	2.75	5.26	4.27	1.69	3.53	1.74	2.44	2.47	4.64	2.64	G	A	46.2		
ss715639339	9	12175377	1.84	0.48	2.07	2.82	5.26	4.75	0.33	4.50	1.46	0.79	0.49	5.89	2.20	T	C	33.2		
ss715647549	11	44651807	7.42	2.67	3.37	3.89	5.40	4.64	1.13	3.74	2.33	1.34	1.83	5.41	4.00	C	T	37.3		
ss715639339	9	12175377	3.62	0.89	2.98	2.10	5.09	0.89	9.20	6.04	2.40	11.28	16.30	10.03	4.75	T	C	11.8	Q1	
ss715647549	11	44651807	7.26	0.83	4.25	5.46	5.36	0.83	7.16	3.71	2.72	9.61	14.47	6.96	5.98	C	T	19.5		
ss715641893	2	10113375	0.82	1.26	1.72	1.74	5.28	5.52	5.90	1.01	1.35	6.54	8.26	0.92	1.49	T	C	43.7	all.315	HG Type 2.5.7
ss715639285	2	33312585	5.92	4.29	2.58	2.65	4.08	6.64	6.42	1.86	1.64	8.14	10.26	2.86	2.80	T	G	38.4		
ss715645573	3	50143102	3.39	5.67	2.86	2.94	3.97	5.67	5.64	2.21	2.52	6.07	9.10	3.39	4.34	C	T	41.1		
ss715645642	9	33052539	0.15	0.05	1.25	1.26	5.32	5.62	6.05	1.19	0.97	6.41	8.45	1.14	0.96	G	T	48.6		
ss715650604	1	41625385	1.11	0.02	1.59	1.62	8.91	5.44	5.21	2.79	1.08	7.58	11.16	5.69	2.46	G	A	39.8	Q1	
ss715651021	1	41732173	1.34	2.48	2.05	2.10	9.10	5.73	5.56	3.32	1.50	8.09	11.84	6.72	3.44	T	C	37.4		
ss715647960	1	41789504	1.12	0.04	1.49	1.51	8.45	5.26	4.96	2.29	0.88	7.29	10.60	4.68	2.01	G	A	38.1		
ss715639285	2	33312585	1.58	0.47	2.47	2.55	5.73	5.68	5.44	2.24	1.67	7.48	11.56	4.56	3.84	T	G	49.5		
ss715640488	7	35740746	6.07	6.17	3.62	3.82	10.35	8.86	9.67	6.09	2.63	18.50	19.60	11.88	6.10	T	C	22.8		
ss715640389	9	12154448	0.68	1.00	3.05	3.18	7.66	8.31	9.83	6.84	2.73	30.85	18.19	11.70	4.86	A	C	11.2		
ss715639339	9	12175377	4.88	2.20	2.98	3.10	8.42	8.40	9.03	6.18	2.05	26.01	18.45	12.06	4.74	T	C	11.9		
ss715641522	11	13037340	0.31	0.06	1.06	1.07	7.20	4.34	4.60	2.18	0.94	5.55	8.39	3.31	1.23	T	C	31.1		
ss715647636	3	3963582	9.29	5.65	2.55	4.38	2.73	2.56	8.18	2.61	2.54	2.78	11.57	2.97	3.12	C	T	11.2	all.315	HG Type 1.2.3.5.6.7
ss715647109	6	27257765	10.60	8.32	3.43	2.92	3.79	3.43	8.55	3.29	3.14	2.76	13.44	4.84	5.14	C	T	9.4		
ss715640509	10	2792311	10.60	5.95	6.19	6.80	1.44	6.19	0.12	7.32	6.76	0.43	0.04	9.40	9.98	T	C	13.0		
ss715639563	11	46491205	6.09	4.15	1.92	2.37	3.33	1.92	7.27	2.62	2.58	4.02	13.38	4.56	4.76	A	G	27.0		
ss715639563	11	46491205	4.11	4.11	4.03	4.30	1.78	4.35	6.06	6.55	5.64	1.52	14.98	15.60	16.27	A	G	9.7	Q1	
ss715639339	9	12175377	1.84	0.48	2.07	2.82	5.26	4.75	0.33	4.50	1.46	0.79	0.49	5.89	2.20	T	C	33.2	all	HG Type 0
			3.62	0.89	2.98	2.10	5.09	0.89	9.20	6.04	2.40	11.28	16.30	10.03	4.75	T	C	11.8	Q1	
			0.33	0.54	2.14	2.18	1.36	0.31	0.33	4.15	1.65	0.56	0.57	6.26	2.83	T	C	33.2	all	HG Type 2.5.7
			4.88	2.20	2.98	3.10	8.42	8.40	9.03	6.18	2.05	26.01	18.45	12.06	4.74	T	C	11.9	Q1	

### Genome-Wide Association Study for Resistance to SCN HG Type 0

**Figure 3:**
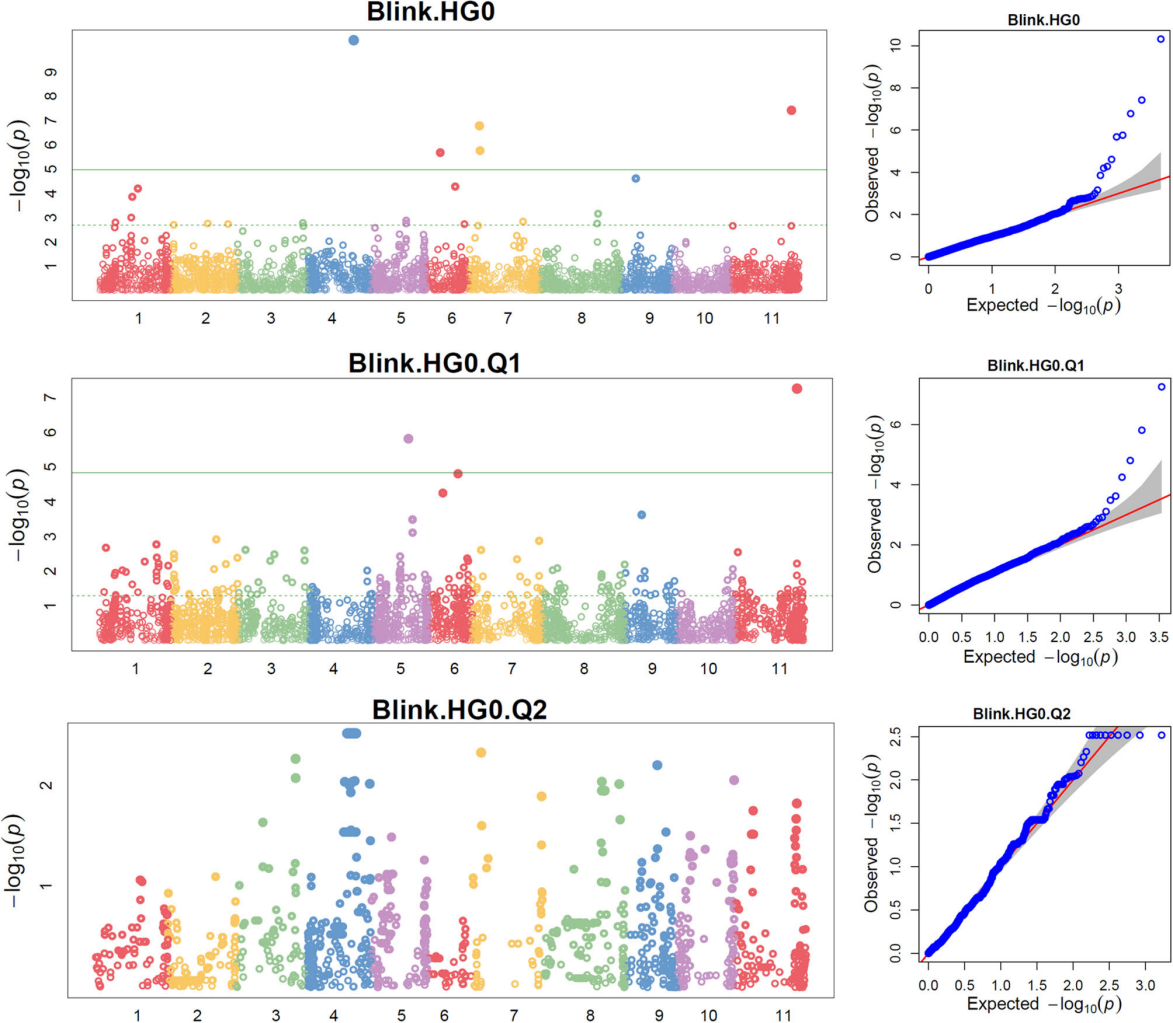
Distributions of Manhattan plot (left side) and QQ-plot (right side) of genome-wide association study (GWAS) for common bean resistance to SCN HG Type 0 (race 6) in all 315 accessions (top), 241 accessions of Q1 population (middle), and 67 accessions of Q2 population (bottom) based on BLINK, where x-axis represents the common bean 11 chromosomes and y-axis represents LOD [–log(*P*-value)] value of each SNP in Manhattan plot, and x-axis represents the Expected LOD [–log(*P*-value] and y-axis represents Observed LOD [–log(*P*-value)] value of each SNP in QQ-plot.

The distributions of the QQ plots between the observed *vs*. expected LOD [−log_10_ (p)] showed a large divergence from the expected distribution (Supplementary Figure 5), indicating there were SNPs associated with the resistance to SCN HG Type 0 in the three association panels. The Manhattan plot showed there were a dozen SNPs with LOD value >4.97 in all.set and Q1 (Supplementary Figure 5), and associated with SCN resistance to HG Type 0. Based on MLM models, a total of 18 SNPs, located on Pv 03, 04, 05, 06, 07, 08, 09, and 11 had LOD > 4.79 in all.set or > 4.84 in Q1 (Supplementary Table 5), associated with the resistance to SCN HG Type 0 (Supplementary Table 5). Among the six models, BLINK had the highest LOD values, and several SNP markers were observed in all.set and Q1 but not in Q2 (Figure 3).

There were several SNPs with LOD > 4.97 in all.set and > 4.84 in the Q1 population in the SMR and GLM models but not in the MLM model (Supplementary Table 5), indicating that there were significant SNP markers, but they were not strongly associated with SCN resistance based on the Tassel tool. However, there were several SNPs with a LOD score > 4.0 or 3.0, indicating there were small-effect QTLs for SCN resistance (Supplementary Table 5). Based on *t*-tests, all 18 SNPs had LOD values > 2.0 (*P* < 0.01) either in all.set, Q1, or Q2 (Supplementary Table 5).

After combining, six SNP markers, ss715640464, ss715650114, ss715647158, ss715649511, ss715639339, and ss715647549, located on chromosomes Pv04, 06, 07, 07, 09, and 11, were associated with resistance to SCN HG Type 0 in all.set (Table 2). The two SNPs, ss715647158 and ss715649511, were located at 7,343,812 bp and 7,759,866 bp, respectively, on Pv07 based on the Pvulgaris v1.0_218 whole-genome reference sequences (Table 2), suggesting that there was a QTL on Pv07 for HG Type 0 resistance. The ss715639339 SNP at 12,175,377 bp on Pv09 and ss715647549 at 44,651,807 bp on Pv11 were observed in both all.set and Q1 for HG Type 0 resistance (Table 2), suggesting the presence of a QTL on each of the two chromosomes.

### Genome-Wide Association Study for Resistance to SCN HG Type 2.5.7

**Figure 4:**
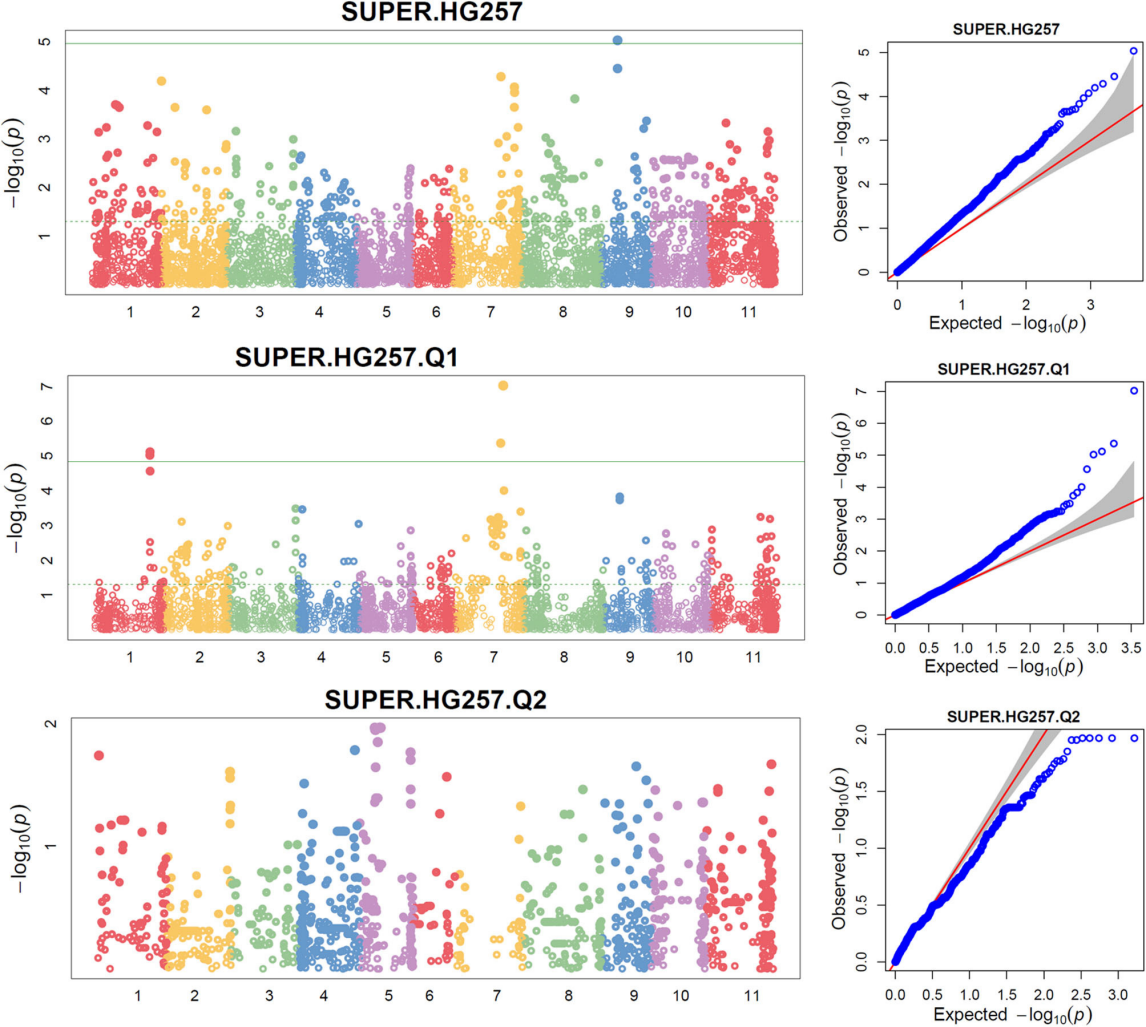
Distributions of Manhattan plot (left side) and QQ-plot (right side) of GWAS for common bean resistance to SCN HG Type 2.5.7 in all 276 accessions (top), 207 accessions of Q1 population (middle), and 62 accessions of Q2 population (bottom) based on SUPER, where x-axis represents the common bean 11 chromosomes and y-axis represents LOD [–log(*P*-value)] value of each SNP in Manhattan plot, and x-axis represents the Expected LOD [–log(*P*-value] and y-axis represents Observed LOD [–log(*P*-value)] value of each SNP in QQ-plot.

The distributions of the QQ plots between the observed *vs*. expected LOD [-log_10_ (p)] showed a large divergence from the expected distribution (Supplementary Figure 6), suggesting there were SNPs associated with resistance to SCN HG Type 2.5.7 in the three association panels. The Manhattan plot showed there were a dozen SNPs with a LOD value >4.97 in all.set (Supplementary Figures 6A,B) and Q1 (Supplementary Figures 6C,D) for resistance to HG Type 2.5.7. A total of 15 SNPs, located on chromosomes Pv01, 02, 03, 07, 09, and 11 had LOD > 4.79 in all.set, or > 4.84 in Q1 (Supplementary Table 6). Among the six models, SUPER had the highest LOD values, and several SNP markers had LOD values greater than the 4.97 significance threshold in all.set, and > 4.84 in Q1, but not in Q2 (Figure 4, Supplementary Table 6).

The TASSEL 5 analysis showed that there were several significant SNPs with a LOD score > 4.97 in all.set and > 4.84 in the Q1 population in the SMR and GLM models but not in the MLM model (Supplementary Table 6). Nevertheless, these markers were not strongly associated with SCN resistance. However, there were several SNPs with a LOD score > 3.0 or 2.5, suggesting there were QTLs for HG Type 2.5.7 resistance with a small effect (Supplementary Table 6). Based on *t*-tests, 14 of the 15 SNPs had a LOD value > 2.0 (*P* < 0.01) either in all.set, Q1, or Q2, (Supplementary Table 6), indicating that the 14 SNPs were associated with resistance to HG Type 2.5.7 at the *P* = 0.01 significance level.

After combining, four SNPs were associated with resistance to the HG Type 2.5.7 in all.set, eight SNPs in Q1, and none in Q2 (Table 2). Among the eight SNPs in Q1, the three SNPs, ss715650604, ss715651021, and ss715647960, were located in the same region of chromosome Pv01, from 41,625,385 bp to 41,789,504 bp, indicating that there was a QTL on Pv01 for HG Type 2.5.7 resistance. The ss715639285 was identified in both all.set and Q1, suggesting that there was a QTL in the 33.3 Mbp region on Pv02 for HG Type 2.5.7 resistance. The two SNPs, ss715640389 and ss715639339, were located in the same region, from 12,154,448 bp to 12,175,377 bp on Pv09, and the two SNPs had very high LOD values (>26) in the *t*-test (Table 2). In addition, a SNP, ss715640488 at 35,740,746 bp on Pv07 and another SNP, ss715641522, at 13,037,340 bp on Pv11 were associated with HG Type 2.5.7 resistance.

### Genome-Wide Association Study for Resistance to SCN HG Type 1.2.3.5.6.7

**Figure 5:**
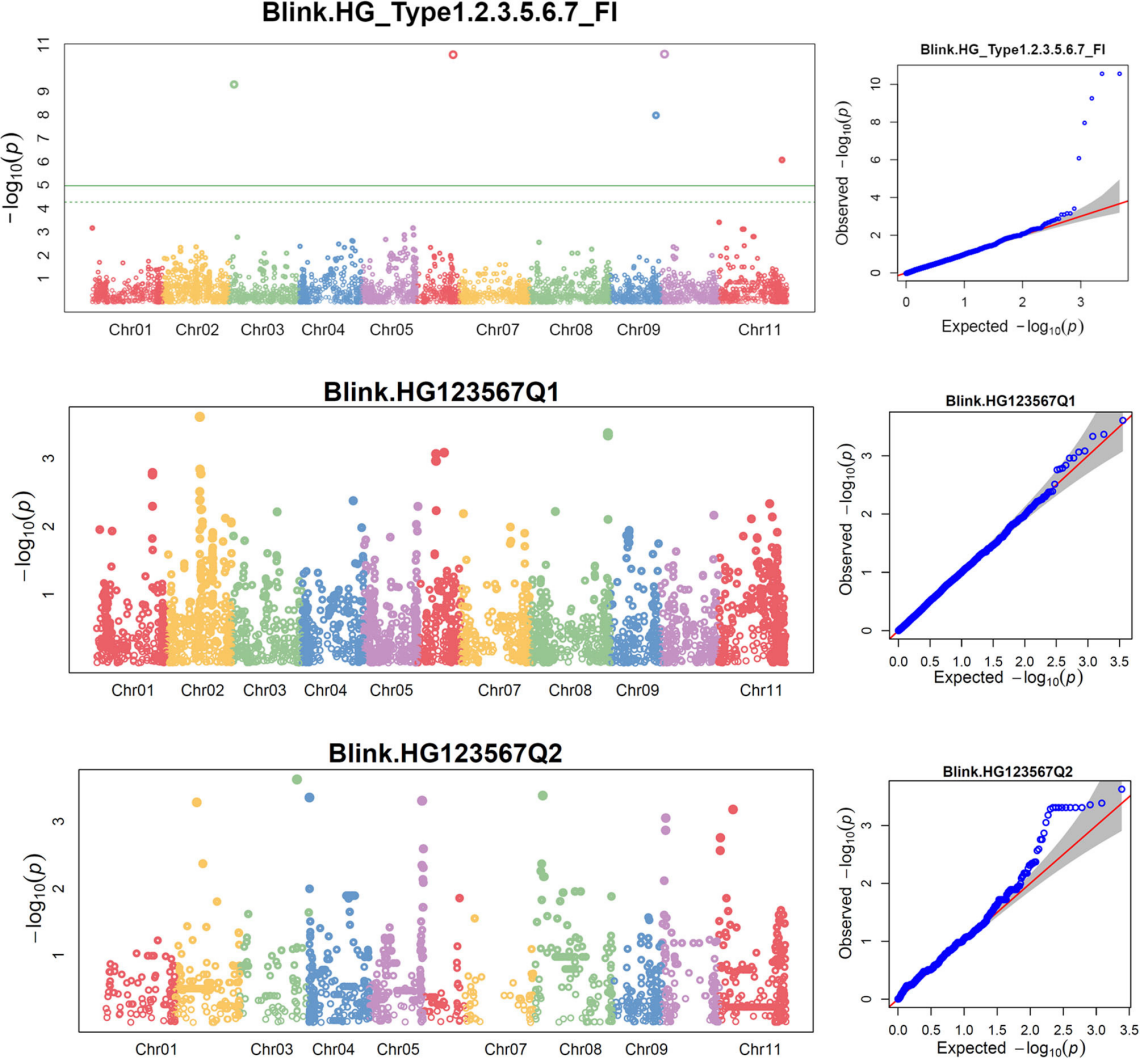
Distributions of Manhattan plot (left side) and QQ-plot (right side) of GWAS for common bean resistance to SCN HG Type 1.2.3.5.6.7 in all 276 accessions (top), 207 accessions of Q1 population (middle), and 62 accessions of Q2 population (bottom) based on BLINK, where the x-axis represents the common bean 11 chromosomes and the y-axis represents LOD [–log(*P*-value)] value of each SNP in Manhattan plot, and x-axis represents the Expected LOD [–log(*P*-value)] and the y-axis represents Observed LOD [–log(*P*-value)] value of each SNP in QQ-plot.

The distributions of the QQ plots between the observed *vs*. expected LOD [–log_10_ (p)] showed a large divergence from the expected distribution (Supplementary Figure 6), indicating there were SNPs associated with resistance to SCN HG Type 1.2.3.5.6.7 in the three association panels. The Manhattan plot showed there were several SNPs with LOD values >4.97 in all.set (Supplementary Figures 7A,B), suggesting there were SNPs associated with SCN resistance to HG Type 1.2.3.5.6.7. Six SNPs, ss715647636, ss715647109, ss715647614, ss715649401, ss715640509, and ss715639563, located on chromosomes Pv 03, 06, 09, 09, 10, and 11, respectively had LOD > 4.79 in all.set (Supplementary Table 7). Among the six models, BLINK had the highest LOD values, and several SNP markers were observed with a significant LOD value > 4.97 in all.set but not in Q1 or Q2 (Figure 5), indicating there were significant SNPs associated with SCN resistance to HG Type 1.2.3.5.6.7 based on the association panel of all.set of 276 accessions. Two additional SNPs, ss715646397 and ss715648134, located on Pv03 and 04, also had LOD values greater than four and were selected as markers for HG Type 1.2.3.5.6.7 resistance in the Q1 population (Supplementary Table 7).

There were only three SNPs that had a LOD score > 4.97 in all.set and one SNP with LOD > 4.84 in the Q1 population, either in SMR, GLM, or MLM models (Supplementary Table 7). However, seven out of the eight listed SNPs had LOD > 3.0 or 2.5 in all.set or Q1, suggesting there were QTLs for SCN resistance with small effects (Supplementary Table 7). The *t*-tests indicated that the eight SNPs had a LOD value > 2.0 (*P* < 0.01) either in all.set, Q1, or Q2 (Supplementary Table 7).

After combining, four SNPs were associated with resistance to SCN HG Type 1.2.3.5.6.7 in all.set, one SNP in Q1, and none in Q2 (Table 2). The four SNP markers in all.set were ss715647636, ss715647109, ss715640509, and ss715639563, located at 3,963,582 bp, 27,257,765 bp, 2,792,311 bp, and 46,491,205 bp on Pv 03, 06, 10, and 11, respectively (Table 2). SNP marker ss715639563 was also identified in Q1 population, increasing the confidence in this SNP as a marker for HG Type 1.2.3.5.6.7 resistance.

### Combining GWAS for Resistance to the Three SCN HG Types

In this study, a total of 40 SNPs were identified as potential SNP markers associated with SCN resistance (Supplementary Tables 5–7) based on the LOD values from the MLM models in GAPIT3 and Tassel 5, after Bonferroni correction. Combining results from the six models (GLM, MLM, SUPER, MLMM, FarmCPU, and BLINK) in GAPIT3, three models (SMR, GLM, and MLM) in TASSEL 5, and *t*-tests among the three association panels (all.set, Q1, and Q2), 6, 11, and 4 SNPs were significantly associated with resistance to HG Type 0, 2.5.7, and 1.2.3.5.6.7, respectively (Table 2). Among them, one SNP, ss715639339, at 12,175,377 bp on Pv09 was associated with the resistance to both SCN HG Types 0 and 2.5.7 (Table 2).

We did not conduct LD analysis for all SNPs in this study. However, the LD heatmaps were drawn, using Haploview for seven genome regions with the eight SNP markers significantly associated with resistance to either SCN HG Type 0, 2.5.7, or 1.2.3.5.6.7 (Supplementary Figure 8), where two LLR genes were also included: Phvul.006G104700 and Phvul.010G018300. The Phvul.006G104700 gene is located on Pv04 in the same LD block as an SNP marker SS715640464 at a distance of only 8.98 Kbp (Supplementary Figure 8A) for HG Type 0 resistance. The gene Phvul.010G018300 is located on Pv10 at a distance of 39.9 Kbp from ss715640509 associated with HG Type 1.2.3.5.6.7 resistance (Supplementary Figure 8E, bottom left).

### Candidate Genes for SCN Resistance

A total of 20 significant GWAS-derived SNPs were selected as markers associated with the resistance to the three SCN HG Types, 0, 2.5.7, and 1.2.3.5.6.7 (Table 2). Candidate gene models were searched within 10, 30, and 50 kb, flanking each of these SNPs. A total of 125, 83, 33, 19, and 8 genes were found at a distance of 50, 30, 10, 5, and 1 Kb, respectively, from the 20 SNPs (Supplementary Table 8) based on the annotations of the common bean genome reference Pvulgaris v1.0_218. Among the 125 genes, five gene models, Phvul.001G158800, Phvul.002G072100, Phvul.006G160700, Phvul.007G080900, and Phvul.009G223200, contained an SNP marker, ss715647960, ss715641893, ss715647109, ss715649511, and ss715645642, respectively, on chromosomes Pv01, Pv02, Pv06, Pv07, and Pv09 (Table 3). Whether these five gene models are related to SCN resistance needs further study.

**Table 3:** Candidate genes for SCN resistance in common bean.

**Gene**	**Chr**	**Start**	**End**	**Arabi-defline**	**SNP**	**Chr**	**Position**	**Distance from the gene start**	**Distance from the gene end**	**Distance from the SNP to the gene**	**Associated SCN HG Type**
Phvul.001G158800	1	41789351	41790573	C2H2 and C2HC zinc fingers superfamily protein	ss715647960	1	41789504	153	−1,069	on the gene	HG257
Phvul.002G072100	2	10112472	10116406	aldehyde dehydrogenase 2B7	ss715641893	2	10113375	903	−3,031	on the gene	HG257
Phvul.004G104700	4	33316658	33320257	disease resistance family protein/LRR family protein	ss715640464	4	33307678	−8,980	−12,579	within 10 kb distance	HG0
Phvul.006G023100	6	10522343	10526782	NAC domain containing protein 42	ss715650114	6	10550456	28,113	23,674	within 30 kb distance	HG0
Phvul.006G160700	6	27256833	27258542	sugar transporter 1	ss715647109	6	27257765	932	−777	on the gene	HG123567
Phvul.007G080900	7	7759099	7762514	Protein of unknown function (DUF677)	ss715649511	7	7759866	767	−2,648	on the gene	HG0
Phvul.009G223200	9	33050989	33058854	ARID/BRIGHT DNA-binding domain-containing protein	ss715645642	9	33052539	1,550	−6,315	on the gene	HG257
Phvul.010G018300	10	2832211	2839756	Leucine-rich repeat protein kinase family protein	ss715640509	10	2792311	−39,900	−47,445	within 50 kb distance	HG123567

The Leucine-Rich Repeat (LRR) gene model Phvul.004G099300 (disease resistance family protein/LRR family protein), located at 33,316,658–33,320,257 bp on Pv04, based on the common bean genome reference Pvulgaris v1.0_218, is located near the SNP marker ss715640464 (distance of 8.98 Kbp), associated with SCN HG Type 0 resistance. Another LRR gene, Phvul.010G018300 (LRR protein kinase family protein) at 2,832,211–2,839,756 bp on Pv10 is close to the SNP marker ss715640509 (distance of 39.9 Kbp). In addition, one NAC-domain gene, Phvul.006G023100 (NAC-domain containing protein 42), is located at 10,522,343–10,526,782 bp on Pv06 was close (~24 Kbp) to the SNP marker ss715650114 (Table 3). Whether the two LRR genes and the NAC-domain gene are related to SCN resistance needs further evaluation.

### Genomic Prediction of SCN Resistance

#### Genomic Prediction With Different Ratios of a Training Set to a Testing Set

**Figure 6:**
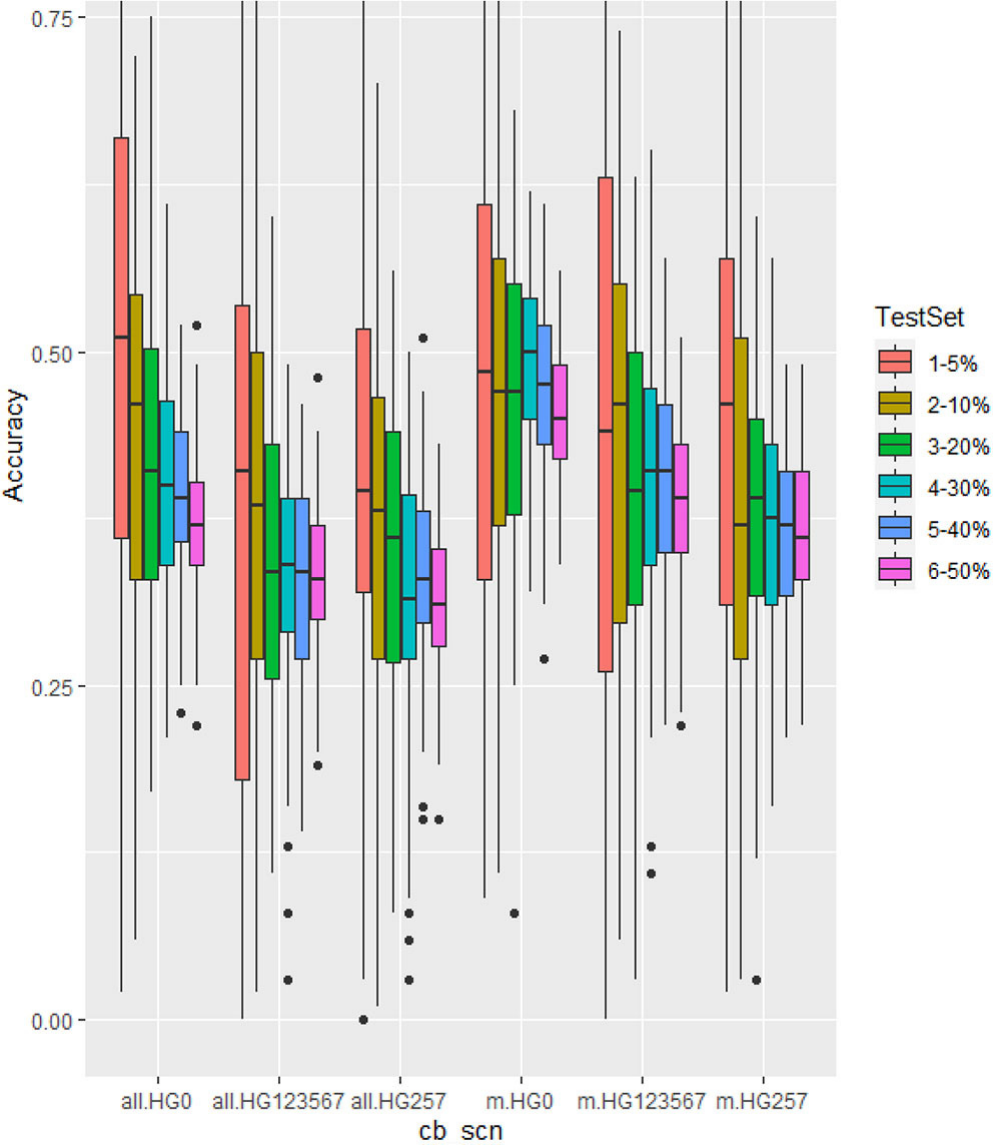
Genomic prediction of six different testing set percentages from 5 to 50% in cross-prediction for resistance to three SCN HG Types, 0, 1.2.3.5,6,7, and 2.5.7 using all 4,654 SNPs (left three groups as all.HG0, all.HG123567, and all.HG257), and 20 associated SNP markers (m.HG0, m.HG123567, and m.HG257) estimated by rrBLUP model.

In this study, GP was performed using six different ratios of training/testing sets, 19:1, 9:1, 4:1, 7:3, and 1:1, expressed as 5, 10, 20, 30, 40, and 50% of a testing set in all.set, containing the 315 common bean accessions for HG Type 0 resistance or 276 accessions for HG Types 2.5.7 and 1.2.3.5.6.7 resistance. The actual sizes of the [training set/testing set] were 299/16, 283/32, 252/63, 220/95, 189/126, and 158/157 for HG Type 0, and 262/14, 248/28, 221/55, 193/83, 166/110, and 138/138 for HG Types 2.5.7 and 1.2.3.5.6.7. The GEBVs and r- values between GEBVs and observed values in the testing set were estimated by six GP models (rrBLUP, gBLUP, Bayes A, Bayes B, BL, and BRR) in cross-prediction for resistance to the three HG Types, 0, 2.5.7, and 1.2.3.5.6.7, using (1) all 4,654 SNPs and (2) 20 associated SNP markers (20 GWAS-derived SNP markers). There were six ratios between training and testing sets, six models, two SNP sets, and three SCN HG types to make a total of 216 combinations. Each combination was run 100 times to calculate GP statistical parameters and r-values. The average r-value (r_Ȳ100_) and its SE from the 100 runs for each combination are listed in Supplementary Table 9 and the 216 averaged r-values (r_Ȳ100_) displayed in charts drawn in MS Excel 2016, grouped by the six sets (5, 10, 20, 30, 40, and 50%) of testing set percentages (Supplementary Figure 9). The r-distribution charts were created by an R-package for the 216 combinations grouped by percentages of a testing set; the r-distributions of the 36 combinations estimated by rrBLUP model are listed in Figure 6. The 108 averaged r-values (r_Ȳ100_) (half of all 216 combinations) for the all.set are listed in Table 4.

**Table 4:** Prediction accuracy (PA) for SCN resistance to three HG Types with six different testing sets (percentages) using all 4,654 SNPs with six genomic prediction models.

**GP model**	**r-value in HG Type 0**							**r-value in HG Type 2.5.7**							**r-value in HG Type 1.2.3.5.6.7**						
	**5%**	**10%**	**20%**	**30%**	**40%**	**50%**	**Average**	**5%**	**10%**	**20%**	**30%**	**40%**	**50%**	**Average**	**5%**	**10%**	**20%**	**30%**	**40%**	**50%**	**Average**
rrBLUP	0.44	0.41	0.41	0.41	0.40	0.37	0.41	0.33	0.36	0.35	0.32	0.33	0.32	0.34	0.30	0.36	0.34	0.33	0.33	0.33	0.33
gBLUP	0.38	0.31	0.30	0.29	0.28	0.27	0.31	0.25	0.31	0.27	0.26	0.24	0.23	0.26	0.11	0.11	0.12	0.10	0.08	0.08	0.10
Bayes A	0.41	0.39	0.39	0.42	0.39	0.39	0.40	0.33	0.40	0.37	0.36	0.35	0.34	0.36	0.31	0.31	0.30	0.30	0.29	0.29	0.30
Bayes B	0.40	0.40	0.38	0.40	0.37	0.36	0.39	0.34	0.35	0.33	0.35	0.33	0.31	0.33	0.32	0.30	0.30	0.31	0.29	0.28	0.30
BL	0.43	0.43	0.43	0.40	0.38	0.38	0.41	0.37	0.33	0.35	0.37	0.35	0.34	0.35	0.27	0.34	0.28	0.29	0.27	0.28	0.29
BRR	0.44	0.41	0.40	0.41	0.39	0.38	0.41	0.38	0.36	0.38	0.35	0.34	0.32	0.36	0.31	0.35	0.34	0.34	0.31	0.31	0.33
Average	0.42	0.39	0.39	0.39	0.37	0.36	0.39	0.33	0.35	0.34	0.34	0.32	0.31	0.33	0.27	0.30	0.28	0.28	0.26	0.26	0.27

The six sets of 5, 10, 20, 30, 40, and 50% of testing set percentages had similar, although not identical averaged r-values across five models except gBLUP with slightly lower r-values (Table 4, Figure 4, Supplementary Figure 9, Supplementary Table 9). The r-value, averaged over six models, was 0.39 for HG Type 0, 0.33 for HG Type 2.5.7, and 0.27 for HG Type 1.2.3.5.6.7. They were 0.40 for HG Type 0,0.35 for HG Type 2.5.7, and 0.31 for HG Type 1.2.3.5.6.7 when averaged from five models, except gBLUP, when using all 4,654 SNPs (Table 4, Supplementary Table 9). This observation suggested that it may be feasible to do GS for SCN resistance in common bean with one of the six sets. The r-value increased to 0.46,0.38, and 0.41, averaged over the six models, and 0.51,0.41, and 0.46, averaged over the five models (except gBLUP) when using only the 20 SNP markers (Supplementary Table 9, Supplementary Figure 9), suggesting that GWAS-derived SNP markers can be used in GS. From Figure 6, the 5% test set had the largest variance, and the 50% test set had the smallest. The PA decreased when the size of the testing set increased. Likewise, the SE values decreased when the test sets increased from 5 to 50% (Supplementary Table 9), indicating that the larger the testing set, the less variable the r-values. However, a small decrease of the r-value was observed as well in most cases when the training/test ratio was 40% or higher.

#### Genomic Prediction With Different SNP Numbers

**Figure 7:**
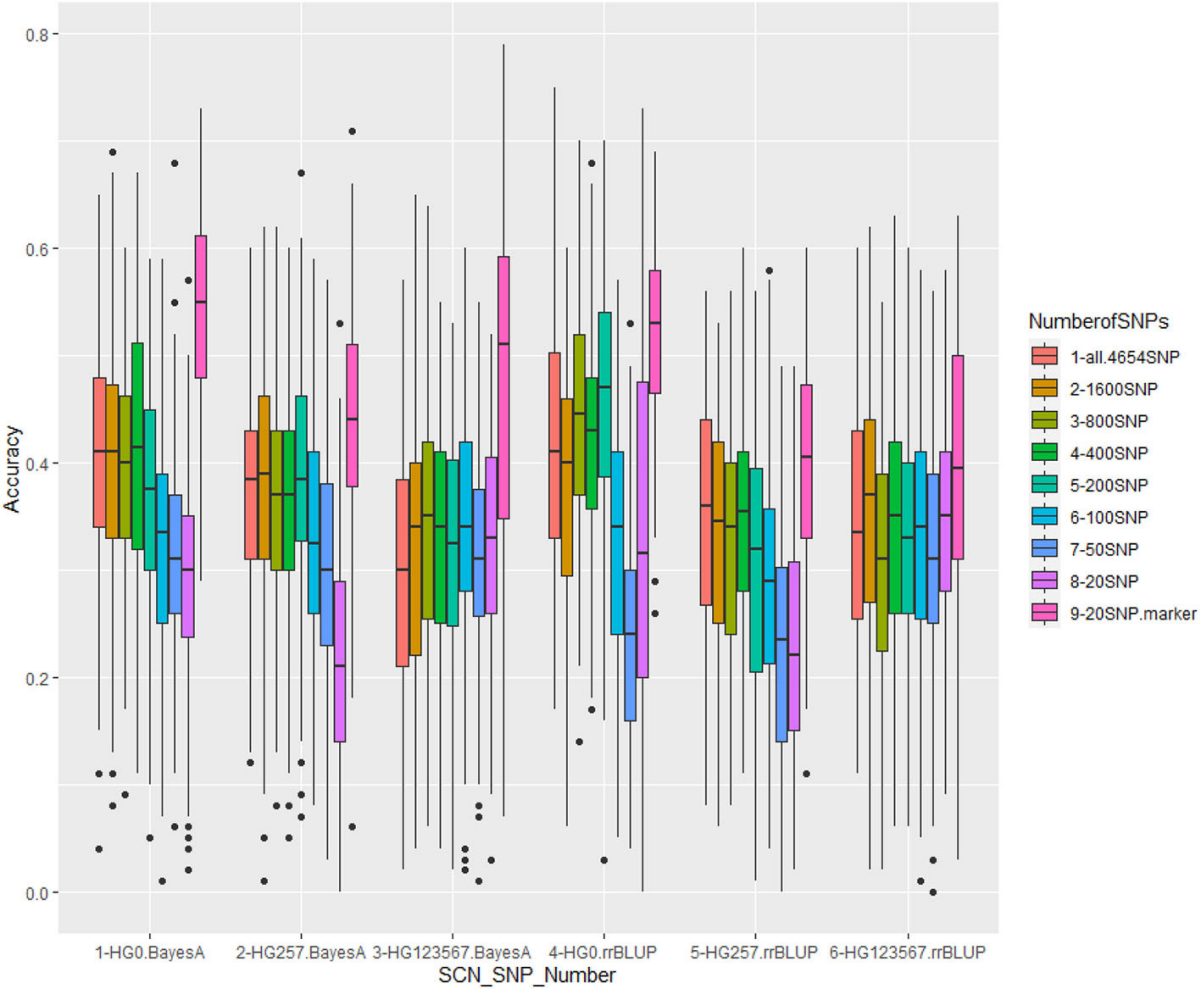
Genomic prediction of nine different SNP numbers from 20 SNPs to all 4,654 SNPs in cross-prediction for resistance to three SCN HG Types, 0, 2.5.7, and 1.2.3.5.6.7 using Bayes A model (left three groups) and rrBLUP model (right three groups).

Genomic prediction was performed with nine different SNP number sets (20, 50, 100, 200, 400, 800, 1,600, and all 4,654 SNPs, plus the 20 GWAS-derived SNP markers) in cross-predictions for resistance to three HG Types, using five GP models: rrBLUP, Bayes A, Bayes B, BL, and BRR. There were 135 combinations for GP analysis, consisting of nine SNP sets, five GP models, and three SCN HG Types. Each combination was run 100 times to calculate GP statistical parameters and r-values. The average r-value (r_Ȳ100_) and its SE from the 100 runs for each combination are presented in the Supplementary Table 10, Supplementary Figure 10. The 27 averaged r-values (r_Ȳ100_) estimated by rrBLUP are presented in Table 5. The 54 r-distribution charts created by ggplots in R-package for r-values, estimated by Bayes A and rrBLUP models, are shown in Figure 7.

**Table 5:** Genomic prediction of nine different SNP number sets from 20 SNPs to all 4,654 SNPs in cross-prediction for resistance to SCN HG Types 0, HG Type 2.5.7, and HG Type 1.2.3.5.6.7 using rrBLUP.

**HG Type**	**4654SNP**	**1600SNP**	**800SNP**	**400SNP**	**200SNP**	**100SNP**	**50SNP**	**20SNP**	**20SNP.marker**	**Average**
HG Type 0	0.41	0.38	0.44	0.42	0.45	0.33	0.22	0.27	0.52	0.38
HG Type 257	0.35	0.34	0.32	0.34	0.30	0.28	0.22	0.21	0.40	0.31
HG Type 123567	0.34	0.34	0.30	0.34	0.33	0.32	0.31	0.34	0.39	0.33

The nine SNP sets had an averaged r-value 0.38 for HG Type 0**,0.3**1 for HG Type 2.5.7, and 0.33 for HG Type 1.2.3.5.6.7 (Table 5, Figure 7, Supplementary Figure 10). The r-values were somewhat decreased a little when 100 or less SNPs were used for HG Type 0 resistance, 200 or less SNPs were used for HG Type 2.5.7 resistance, but did not decrease for HG Type 1.2.3.5.6.7 resistance, indicating that sets of more than 200 SNPs can be used for GS. The set of the 20 SNP markers had the highest averaged r-values in all five models for the three HG Type resistances (Figure 7), indicating that the 20 associated SNP markers can be used to do GS for SCN resistance selection as well.

#### Genomic Selection in Three Association Panels

Genomic prediction was performed in the three association panels (all.set, Q1, and Q2) with six different testing set sizes from 5 to 50% in cross-prediction for resistance to the three HG Types, using the rrBLUP model (54 combinations). Each combination was run 100 times to estimate GEBVs and r-values. The average r-value (r_Ȳ100_) and its SE from the 100 runs for each combination are listed in Supplementary Table 11, and the r-charts are shown in Supplementary Figure 11.

For the HG Type 0 resistance, all r-values are similar among the three sets (all.set, Q1, and Q2) across six testing sets with averaged 0.41, 0.41, and 0.38, respectively (Supplementary Table 11, top). For HG Type 2.5.7 and 1.2.3.5.6.7 resistance, all.set and Q1 had similar r-values, but Q2 had much lower r-values (Supplementary Figure 11). The 5% of the “Testing set” had the largest variability, and the 50% had the lowest SE value, and PA decreased when the “Testing set” percentage increased (Supplementary Table 11).

#### Genomic Prediction Comparisons Among All SNPs, SNP Markers, and the Random SNP Set

Genomic prediction was performed for three SNP sets (all 4,654 SNPs, 20 GWAS-derived SNP markers, and 20 random SNPs) in cross-prediction for resistance to three HG Types, using eight GP models (rrBLUP, gBLUP, Bayes A, Bayes B, BL, BRR, RF, and SVM) (72 combinations). Each combination was run 100 times to estimate GEBVs and r-values. The average r-value (r_Ȳ100_) and SE from the 100 runs for each combination are presented in Supplementary Table 12, and the r-charts are also showed in Supplementary Figure 12.

The set of 20 GWAS-derived SNP markers had the highest r-values across the eight models for resistance to either HG Type 0, 2.5.6, or 1.2.3.5.6.7, suggesting that the GWAS-derived SNP markers will be more effective for GS than the random 20-SNP sets (Supplementary Table 12, Supplementary Figure 12). The set of “random 20 SNPs” had the lowest r-values, suggesting that using more SNPs would increase the selection effectiveness in GS.

#### Genomic Prediction Using Different Models

Eight GP models (rrBLUP, gBLUP, Bayes A, Bayes B, BL, BRR, RF, and SVM) were used to conduct GP for resistance to the three HG Types. The five GP models (rrBLUP, Bayes A, Bayes B, BL, and BRR) had similar r-values, but the gBLUP model had the lowest r-values for resistance to either HG Type 0, 2.5.7, or 1.2.3.5.6.7 (Supplementary Figure 13C).

Based on the results from six different testing sets (percentages) in 315 common bean accessions (Table 4, Supplementary Table 9), the six GP models (rrBLUP, gBLUP, Bayes A, Bayes B, BL, and BRR) had similar averaged PA **(**0.41, 0.31, 0.40, 0.39, 0.41, and 0.41) for resistance to HG Type 0; lower but similar PA (0.34, 0.26, 0.36, 0.33, 0.35, and 0.36) for HG Type 2.5.7 resistance; and the lowest PA (0.33, 0.10, 0.30, 0.30, 0.29, and 0.33) for HG Type 1.2.3.5.6.7 resistance. When the set of 20 significant SNP markers was used, the averaged PA of the six models increased for resistance to all of the three HG Types (Supplementary Table 9, bottom half).

Based on the results from the nine different SNP number sets from 20 SNPs to all 4,654 SNPs in cross-prediction for resistance to the three HG Types (Supplementary Table 10, Supplementary Figure 11), the five GP models (rrBLUP, Bayes A, Bayes B, BL, and BRR) had averaged PA, 0.38, 0.38, 0.36, 0.38, and 0.38, respectively, for resistance to HG Type 0;0.31, 0.35, 0.31, 0.34, and 0.35 for HG Type 2.5.7 resistance; and 0.33, 0.34, 0.30, 0.34, and 0.34 for HG Type 1.2.3.5.6.7 resistance.

Based on the three SNP sets (all 4,654 SNPs, 20 significant SNP markers, and 20 random SNPs) used in cross-prediction, the eight GP models, rrBLUP, gBLUP, Bayes A, Bayes B, BL, BRR, RF, and SVM, had averaged PA values of 0.38, 0.20, 0.41, 0.35, 0.42, 0.40, 0.35, and 0.39, respectively, for resistance to HG Type 0;0.31, 0.20, 0.34, 0.28, 0.34, 0.34, 0.29, and 0.32 for HG Type 2.5.7 resistance; and 0.36, 0.11, 0.37, 0.32, 0.38, 0.39, 0.33, and 0.30 for HG Type 1.2.3.5.6.7 resistance (Supplementary Table 12, Supplementary Figure 12).

Overall, sets of 400 SNPs or more for GP had similar GS efficiency (r-value) for resistance to either HG Type 0, 2.5.7, or 1.2.3.5.6.7. The set of 20 significant SNP markers for GP had the highest r-value for GP (Supplementary Figure 13A). The six sets of different sizes from 5 to 50% had similar r-values (Supplementary Figure 13B). Except for the gBLUP model, which had a lower r-value for GP, all other seven models had similar PA (Supplementary Figure 13C). The averaged r-values were 0.40 for HG Type 0 resistance, 0.34 for HG Type 2.5.7, and 0.32 for HG Type 1.2.3.5.6.7 (Supplementary Figure 13D), indicating that we can use one of the seven GP models to conduct GS. Each model provided similar selection efficiency for SCN resistance.

#### Genomic Heritability (GH)

In this study, the GH was estimated by the rrBLUP model for resistance to the three SCN HG Types, 0, 2.5.7, and 1.2.3.5.6.7 (Supplementary Table 13, Supplementary Figure 14). As we did for GP estimations, the GH was estimated, using six different ratios of the training set: the testing set 19:1, 9:1, 4:1, 7:3, and 1:1, as 5, 10, 20, 30, 40, and 50% of the testing set in the GWAS panel with (1) all 4,654 SNPs (top in both Supplementary Table 13, Supplementary Figure 14), (2) 20 GWAS-derived SNP markers (middle), and (3) nine different SNP number sets from 20 SNPs to all 4,654 SNPs (bottom) in cross-prediction. The averaged GH was 22.4, 12.1, and 5.4% for three HG Types, respectively, in all 4,654 SNPs; 28.1, 22.1, and 6.1% in 20 SNP markers; and 13.7, 10.5, and 3.2% in the nine different SNP number sets from 20 SNPs to all 4,654 SNPs in cross-prediction. The results showed that GH was highest for resistance to HG Type 0, middle for HG Type 2.5.7, and lowest for HG Type 1.2.3.5.6.7, and the GWAS-derived 20 SNP marker set had higher GH (Supplementary Table 13).

### Genetic Diversity Analysis for the SCN-Resistant Germplasm Accessions

There were 47 resistant accessions with FI < 20.0 for resistance to HG Type 0 (Supplementary Table 1). Among the 47 accessions, 10 had FI > 30.0 for resistance to HG Type 2.5.7, although they had FI values <20.0 for resistance to both HG Type 0 and 1.2.3.5.6.7. These 10 accessions were not recognized as broadly resistant lines and were dropped from further genetic diversity analysis. Among the 37 accessions, 27 accessions were originally collected from Mexico, two from Colombia, three from Costa Rica, two from Ecuador, one from Guatemala, and two from Peru (Table 1) indicating that the SCN resistance was mainly distributed among Mesoamerican accessions in this study.

**Figure 8:**
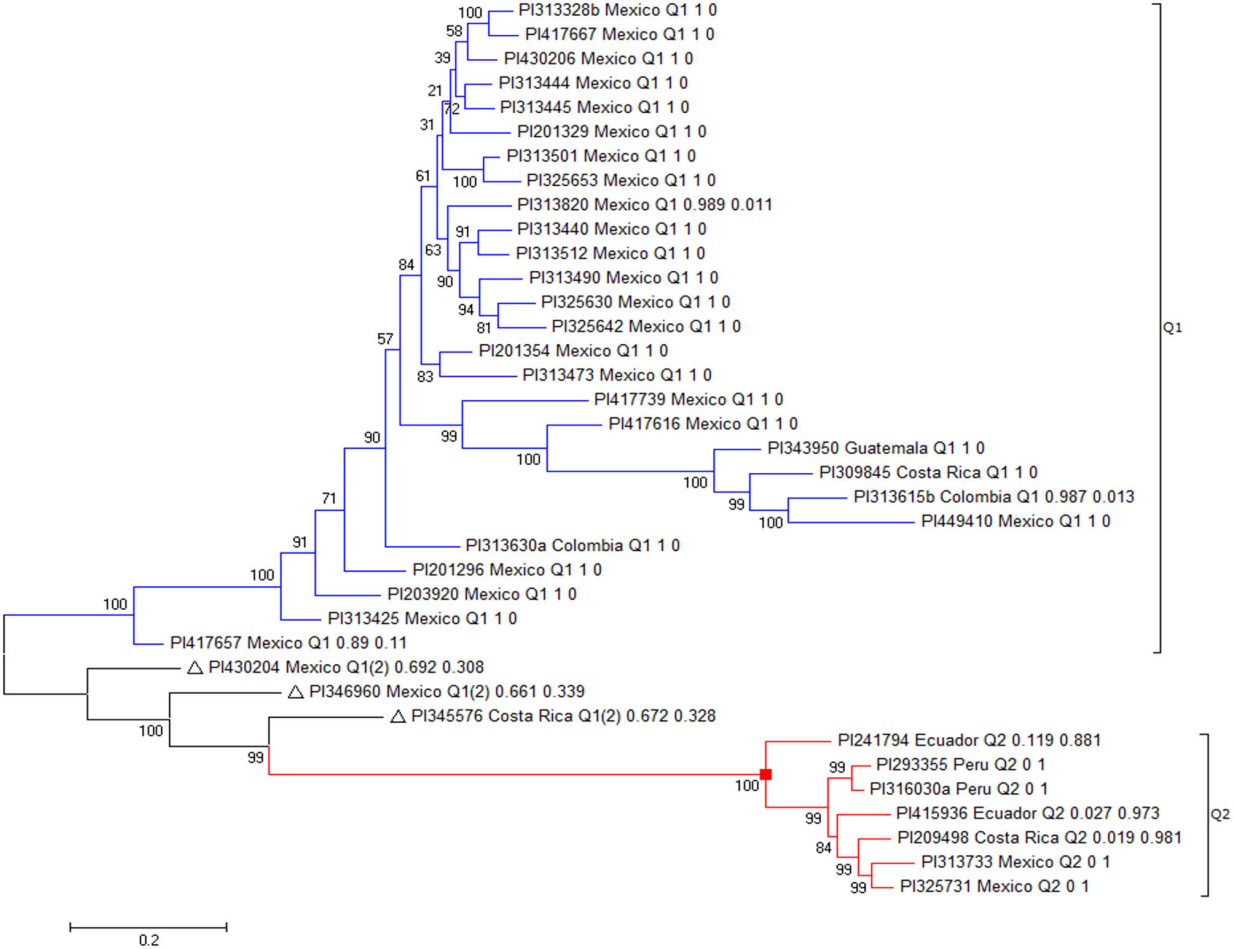
The phylogenetic tree created by the Maximum Likelihood (ML) method from MEGA 7 in 37 common bean germplasm accessions that were resistant to all three SCN HG Types 0, 2.5.7, and 1.2.3.5.6.7.

The 37 accessions formed two clusters as I and II (Figure 8, Table 1). Group I consisted of 27 accessions, which were mainly from Mexico, plus two from Colombia, one from Costa Rica, and one from Guatemala. All of the 27 accessions also belonged to Cluster Q1, based on the population structure and genetic analyses of all 315 accessions. Group II had 10 accessions, including four from Mexico, two from Costa Rica, two from Ecuador, and two from Peru (Figure 8, Table 1). Among the 10 accessions in II, seven belonged to Q2, with a membership coefficient >70%, and three to Q1 or Q1(2) with a membership coefficient of 30%. The latter three accessions, PI430204, PI346960, and PI345576, had Q1 membership coefficients > 66% based on the population structure and genetic analysis in all 315 accessions. In this phylogenetic tree of the 37 accessions (Figure 8), the three accessions, PI430204, PI346960, and PI345576, were clustered to group II but diverged from the cluster. The three accessions plus PI417657 more likely belonged to a subpopulation between clusters I and II, indicating the four accessions combined genetic backgrounds of both clusters (I and II) and the two subpopulations of common bean based on STRUCTURE 2.3.4 analysis.

## Discussion

### Genetic Diversity and Population Structure

In this study, the common bean population structure was examined among 315 common bean germplasm accessions belonging to the USDA *P. vulgaris* core collection, using the Markov Chain Monte Carlo iterations in STRUCTURE 2.3.4. The 315 accessions can be divided into two larger populations (Q1 and Q2 clusters) or into three subpopulations (G1 to G3 plus admixture) (Figure 2, Supplementary Figure 3, Supplementary Table 1).

Based on the two broader populations (Q1 and Q2) in the core collection, Q1 was the larger cluster with 77% (241/315) of accessions and consisted of germplasm mainly from Mexico (145), Guatemala (25), Colombia (20), Costa Rica (13), Nicaragua (12), El Salvador (11), and Honduras (7), with 60, 10, 8, 5, 5, 5, and 3%, respectively (Supplementary Table 14). Q2 consisted of germplasm mainly from Mexico (15), Colombia (15), Peru (14), and Ecuador (11), with 22, 22, 21, and 16%, respectively (Supplementary Table 14). Most of the germplasm accessions from Central America, including Nicaragua (92.3%), Mexico (89.0%), Guatemala (83.3%), El Salvador (84.6%), Costa Rica (76.5%), and Honduras (77.8%) belonged to Q1; most accessions from South America, including Bolivia (only one accession), Peru (82.4%), and Ecuador (68.8%) belonged to Q2; and Colombia accessions belonged to both Q1 and Q2 with 57.1% to Q1 and 42.9% to Q2 (Supplementary Table 13).

Common bean consists of two geographic, diverged gene pools, namely the Andean and Middle American pools (Gepts and Bliss, 1985; Gepts et al., 1986, 2019; Koenig and Gepts, 1989; Koinange and Gepts, 1992; Beebe et al., 1997, 2000; Blair et al., 2009, 2012; Kwak and Gepts, 2009; Bitocchi et al., 2012, 2013; McClean et al., 2012; Schmutz et al., 2014; Campa et al., 2018; Kuzay et al., 2020). The analysis confirmed the presence of two populations (two clusters) among these 315 accessions but notes that the germplasm accessions from Nicaragua, Mexico, Guatemala, El Salvador, Costa Rica, Honduras, Colombia, Ecuador, and Peru include the members of both clusters (populations), indicating that both gene pools existed in these countries.

Based on the three clusters (populations G1 to G3) in the 315 accessions, G1 had accessions mainly from Mexico (32 accessions), Colombia (13), Costa Rica (12), Nicaragua (11), and El Salvador (8), with 33, 13, 12, 11, 10, and 8%, respectively (Supplementary Table 13). G2 consisted of the accessions mainly from Mexico (111) and Guatemala (10), with 80 and 7%, respectively (Supplementary Table 13). G3 came from Mexico (15), Columbia (15), Peru (13), and Ecuador (11), with 23, 22, 20, and 17%, respectively (Supplementary Table 13). Besides, 14 accessions (4%) of the panel were admixed (Supplementary Table 13). For each country, most of the germplasm accessions from the United States (only one accession), Nicaragua (84.6%), Costa Rica (70.6%), El Salvador (61.5%), and Honduras (55.6%) belonged to G1. Most Mexico accessions (68%) belonged to G2; and most accessions from Peru (77%), Ecuador (69%), and Bolivia (only one accession) belonged to G3. The accessions from Guatemala and Colombia belonged to three populations (Q1, Q2, and Q3); 23% of accessions from Guatemala were admixed (Supplementary Table 13). The three Q populations matched those in the report by Kuzay et al. (2020) when K = 3 (Supplementary Table 1). Furthermore, nearly half of the resistant accessions in this core collection belonged to the Middle American gene pool and the Durango ecogeographic race within this gene pool, although some resistant accessions were also identified in race Mesoamerica of the Middle American gene pool and races Nueva Granada and Peru of the Andean gene pool (Supplementary Table 15). Based on these results, we used the three Q-matrices for GWAS in all.set of the 315 accessions to identify SNP markers associated with SCN resistance in this study.

### Genome-Wide Association Study and SNP Marker Identification for SCN Resistance

In this GWAS study, six, 11, and four SNPs were identified to be associated with resistance to HG Types, 0, 2.5.7, and 1.2.3.5.6.7, respectively (Table 2). The six SNPs for HG Type 0 resistance were newly identified markers for resistance to HG Type 0 (race 6) based on their location on chromosomes (Table 2). However, in the region of the two markers, ss715647158 and ss715649511 on Pv07, Jain et al. (2019) also reported an SNP marker ss715648793 (Supplementary Table 16) in the region, further validating a QTL in this region for HG Type 0 resistance. The SNP marker, ss715647549, was significantly associated with HG Type 0 resistance in two association panels, all.set, and Q1 (Table 2), and Jain et al. (2019) also reported six SNPs nearby (Supplementary Table 16), suggesting that there is a QTL on Pv11 for resistance to HG Type 0.Near ss715640464on Pv04 (distance of 8.98 Kbp), a gene model Phvul.004G104700 of the disease resistance family protein/LRR family protein was found (Table 3) in the same LD region (Figure 8), suggesting that Phvul.004G104700 may be associated with the HG Type 0 resistance, but this observation needs to be validated.

For the 11 SNPs with resistance to HG Type 2.5.7, three are located on Pv01, two on Pv02, one on Pv03, one on Pv07, three on Pv09, and one on Pv11 (Table 2). The 11 SNPs are newly identified markers for resistance to HG Type 2.5.7. However, at the ss715639285 region on Pv02 and the ss715645642 region on Pv09, Jain et al. (2019) reported associated SNP markers for HG Type 0 resistance (Supplementary Table 16) and Wen et al. (2019) reported a close SNP marker on Pv09 for resistance to HG Type 2.5.7 resistance (Supplementary Table 16), suggesting that there are QTLs in the regions for SCN resistance. Further studies are needed to validate the broad resistance to multiple HG Types associated with these SNP markers.

The four SNPs with resistance to HG Type 1.2.3.5.6.7 were located on Pv03, 06, 10, and 11 (Table 2), and they are newly identified in this study. However, close to the ss715647109 region on Pv06, Jain et al. (2019) reported an SNP marker, ss715645673, associated with HG Type 0 resistance (Supplementary Table 16), indicating there may be a QTL in the region, but whether the QTL is associated with resistance to the two different HG Types needs to be further validated. Another SNP, ss715639563 at 46,491,205 bp on Pv11 for HG Type 1.2.3.5.6.7 resistance (Table 2), was close (distance ~1.84 Mbp) to ss715647549 at 44,651,807 bp, suggesting a QTL existed in the region, but whether this QTL is associated with resistance to both HG Types needs further study. However, based on the LD analysis (Supplementary Figure 8F, bottom right), the two SNPs, ss715647109 and ss715647549, are located in two different LD regions, suggesting that there are different genes or alleles for resistance to HG Type 0 and 2.5.7.

One SNP, ss715639339 at 12,175,377 bp on Pv09 was associated with both HG Type 0 and HG Type 2.5.7 resistance in two association panels, all.set, and Q1 (Table 2). Another SNP, ss715640389, at 12,154,448 bp on Pv09 was associated with resistance to HG Type 2.5.7 (Table 2). The two SNPs are very close to each other (within 20.929 Kbp) and located in the same LD region (Supplementary Figure 8C), suggesting that a QTL exists for SCN resistance, but further studies are needed to determine whether the QTL is associated with resistance to the two different HG Types.

So far, there are only two GWAS reports for SCN resistance in common beans (Jain et al., 2019; Wen et al., 2019). Wen et al. (2019) conducted GWAS in 363 accessions of the USDA common bean core set for resistance to SCN HG types 2.5.7 and 1.2.3.5.6.7, using 84,416 SNPs obtained with GBS. They found five SNPs on Pv01 and one on Pv09, associated with resistance to HG Type 2.5.7, and only one SNP on Pv07, associated with HG Type 1.2.3.5.6.7 resistance. The five SNP markers with resistance to HG Type 2.5.7 were located at 10,061,925 bp, 18,388,378 bp, 18,388,392 bp, 18,388,403 bp, and 18,388,408 bp on Pv01 of the *P. vulgaris* G19833 Pvulgaris v1.0 reference sequence (Schmutz et al., 2014), with *P*-value from 1.02 × 10^−6^ to 4.94 × 10^−6^, and another one on Pv09 at 35,068,146 bp with *P*-value 1.80 × 10^−6^. The SNP marker for resistance to HG Type 1.2.3.5.6.7 was located at 44,761,605bp on Pv07. We used the SCN phenotypic data from the report by Wen et al. (2019), but a different set of SNPs in BARCBean6K_3 BeadChips (Song et al., 2015) to redo the GWAS analysis. We did not identify the same SNP markers but identified the SNP markers in the same regions for resistance to SCN HG Type 2.5.7, but not for HG Type 1.2.3.5.6.7 resistance (Table 2, Supplementary Tables 6, 7). The two SNPs, ss715640034 and ss715639810, located at 18,874,808 bp and 20,450,707 bp on Pv01 and two SNPs, ss715645642 and SS71549401 located at 33,052,539 bp and 33,956,905 bp on Pv09 (Table 6) were located in similar regions reported by Wen et al. (2019), suggesting that there are QTLs for HG Type 2.5.7 resistance in these regions.

Jain et al. (2019) conducted GWAS in 317 accessions of USDA common bean core collection with SCN HG Type 0 and found 14 significant SNP markers on Pv 04, 05, 06, 07, 08, 10, and 11 in the Middle American subpopulation (179 accessions) and 23 SNP markers on Pv 01, 02, 07, 08, 09, and 11 for the Andean subpopulation (138 accessions). However, we could not find any of the 37 SNPs with LOD values greater than the significant threshold values of 4.97 in all.set, 4.84 in Q1, and 4.52 in Q2 for the resistance to HG Type 0, 2.5.7, or 1.2.3.5.6.7, respectively (Supplementary Table 16). Nevertheless, 11 of the 37 SNPs had at least one LOD value >3.0 from GAPIT3 or TASSEL 5 and, also, a LOD score > 3.0 in *t*-tests for resistance to the population of HG Type 0 (Supplementary Table 17a). We did not retain them as significant associated SNP markers because each of the 11 SNPs did not have LOD values greater than the significant threshold, even <3.0 in any MLM model, although they may be associated with the resistance to HG Type 0 with a minor effect (Supplementary Table 17a). In addition, we observed nine and 10 SNPs with LOD values >3.0 in one or more models and *t*-tests as well (Supplementary Tables 17b,c), suggesting these SNPs have minor effects for resistance to either HG Type 2.5.7 or 1.2.3.5.6.7.

### Candidate Gene Model

Wen et al. (2019) reported three gene models, PHAVU_001G248000g (amino acid transporter), PHAVU_001G247900g (α-SNAP protein), and PHAVU_001G247700g (wound inducible protein 12), located at 50,653,407–50,655,828 bp, 50,646,068–50,650,097 bp, and 50,629,261–50,630,123 bp respectively, on Pv01 of common beans to be associated with resistance to HG Type 2.5.7, which corresponded to three gene models in the *rhg1* region of soybean chr18 with 91%, 94%, and 88% identities. However, Wen et al. (2019) did not report any associated SNP marker in a 50 Mbp region of chromosome Pv01; the closest gene model was located at 18,388,408 bp, which was 32 Mbp distance away from the three genes. The data of resistance to SCN HG Type 2.5.7 from Wen et al. (2019) did not confirm the *rhg1* in soybean existed in common beans for their study. Jain et al. (2019) also reported several candidate genes on Pvulgaris v1.0 Pv01 and Pv08, which had high similarity to the three genes of *rhg1* of soybean for SCN resistance, but they did not report any significant SNP marker located in the candidate gene regions, which were associated with the resistance to HG Type 0. Thus, their study could not confirm either that there is *rhg1* or *Rhg4* resistance in common beans. From the study, an SNP marker, ss715645939, was associated with HG Type 2.5.7, which was located at 48,772,176 bp on Pvulgaris v1.0 Pv01, at a distance of around 1.9 Mbp from the three *rhg1* paralog genes in common beans (Supplementary Table 6). The low LOD values of the SNP marker (LOD < 4 in all six MLM models and 5.0 in GLM and 5.12 in SMR, Supplementary Table 6) cast doubt about resistance to SCN HG Types at this location.

From this study, two LRR gene models, Phvul.004G099300 and Phvul.010G018300 were identified as candidates for SCN resistance. Phvul.004G099300 (disease resistance family protein/LRR family protein) at 33,316,658–33,320,257 bp on Pv04 was associated with HG Type 0 resistance, and Phvul.010G018300 (LRR protein kinase family protein) at 2,832,211–2,839,756 on Pv10 was associated with resistance to HG Type 1.2.3.5.6.7 (Table 3). However, the LRR gene in the *rhg1* region on chr 18 in soybean was not involved in SCN resistance (Mitchum, 2016). Further studies are needed to validate whether the two genes are responsible for the SCN resistance in common beans.

### Genomic Prediction

Genomic prediction accuracy, using the Pearson's correlation coefficient (r) between the GEBV and the observed values, has been the main parameter to measure the performance of GS (Jarquin et al., 2014, 2016; Zhang J. P. et al., 2016; Qin et al., 2019; Ravelombola et al., 2019, 2020, 2021; Wen et al., 2019; Ali et al., 2020; Keller et al., 2020). The PA is affected by several factors, such as the trait itself with its heritability, marker number, and the marker associated with the trait, and is also affected by GS models, marker density, the level of LD, QTL number, the population size, and the relationship between training population and testing population (Jarquin et al., 2016; Ali et al., 2020; Keller et al., 2020). In this study, five scenarios were tested for genomic PA: (1) different ratios of the training set and the testing set (validation set), (2) different SNP numbers, (3) three association panels, (4) the use of GWAS-derived significant SNP markers, and (5) different GP models for resistance to three SCN HG Types.

In this study, GP was performed, using six different ratios of the training set: the testing set 19:1, 9:1, 4:1, 7:3, and 1:1, as 5, 10, 20, 30, 40, and 50% of the testing set in the panel. The six tests showed similar PA (averaged r-values). A small decrease of the r-value was observed in most cases with testing sets of 40% or higher. But the 5% “Testing set” (19:1 in the training set: the testing set) had the largest variance, and 50% had the smallest. The averaged r-values decreased from 5 to 50% (Table 4, Supplementary Tables 9, 11, and Supplementary Figures 6, 9). The study showed that 10, 20, and 30% of the testing set size (as the same 9:1, 4:1, and 7:3 of the training set: the testing set) are good to be used in GS for HG Type resistance in common beans. Keller et al. (2020) reported that the training set of <30% could reduce PA due to an insufficiently sized training set that resulted in overfitting of the model; they also reported that a training set > 80% can lead to large variation between cross-validations due to an excessively small validation set. The results showed similar trends but 10% of the testing set size (i.e., training set size = 90%) was acceptable to GS. Ravelombola et al. (2021) reported that the average GS accuracy was similarly based on the r-values at 2-fold [training set: testing set (validation set) = 1:1], 3-fold, 4-fold, 5-fold, 6-fold, 7-fold, and 8-fold cross-validation for growth habit, flowering time, and a grain yield in a multi-parent advanced generation intercross (MAGIC) cowpea population under drought condition, but a slightly higher averaged r-value was observed in 7-fold cross-validation for 100-seed weight, perhaps associated with the higher heritability of seed weight (Nienhuis and Singh, 1988).

In this study, GP was also performed with nine different SNP number sets from 20 to all 4,654 SNPs in cross-prediction for resistance to three HG Types, using five GP models: rrBLUP, Bayes A, Bayes B, BL, and BRR (Table 5). PA decreased when 100 or less SNPs were used for HG Type 0 resistance and when 200 or less SNPs were used for HG Type 2.5.7 resistance, but PA did not decrease for HG Type 1.2.3.5.6.7 resistance (Table 5, Figure 7, Supplementary Table 10, Supplementary Figure 10). Overall, the results suggest that > 200 SNPs should be used for GS. Wen et al. (2019) reported the average PA estimated by cross-validation was 0.52 and 0.41 for SCN HG Type 2.5.7 and HG Type 1.2.3.5.6.7, respectively, when 5,000 SNPs or more were used and showed a decrease when 1,000 SNPs were used. In most of the reports, the smaller the number of SNPs used, the lower the PA was (Jarquin et al., 2014, 2016; Zhang J. P. et al., 2016; Wen et al., 2019; Ali et al., 2020). Zhang J. P. et al. (2016) estimated PA (r-value) of seed size based on 309 soybean accessions and reported r = 0.85 when 2,000 SNPs or 31,045 SNPs were included; r = 0.8 when 1,000 SNPs or 500 SNPs were used.

In this study, using GWAS-derived SNP markers led to the highest GP accuracy for resistance to all three SCN HG Types (Supplementary Tables 9, 12, Supplementary Figure 12). Ali et al. (2020) estimated the prediction accuracy of various GS models on yield and yield-related traits in wheat; they reported that the GWAS-derived markers improved PA in most cases. Zhang J. P. et al. (2016) conducted GWAS and identified 48 SNPs on 12 chromosomes associated with soybean seed size. Based on GWAS, they reported that the r-values ranged from 0.64 to 0.74 when 5, 10, and 15 of the 48 SNP markers were used, which were 25% higher than those calculated from the same number of randomly selected SNPs. Qin et al. (2019) reported that the average correlation coefficient (r) among 15 amino acids between the observed values (each amino acid content) and the GEBVs predicted ranged from 0.18 to 0.61 when all 23,279 SNPs were used, from 0.45 to 0.68 when 231 SNP markers, associated with one or more amino acid from GWAS were used; and 0.33 to 0.54 when only the associated SNP markers with the specific amino acid content were used, using RR-BLUP in rrBLUP software. Spindel et al. (2016) developed a GS model (GS + *de novo* GWAS) that combines RR-BLUP with GWAS-derived-markers, which were fitted as fixed effects on the RR-BLUP training data and found that this new model outperformed other models, RR-BLUP, Bayesian LASSO (BL), Reproducing Kernel Hilbert Spaces (RKHS) and RF, and multiple linear regression (MLR) for a variety of traits in multiple environments. Thus, using GWAS-derived SNP markers to perform GS is an approach combining MAS and GS that can be used in the real-world breeding program, although the predictive ability may be biased, using SNP markers from GWAS to predict the GEBVs in the same GWAS panel. The real GP will be lower if conducting predictions in other panels with different individuals. We have tested many traits in several crops and find it is a practical approach to do genome breeding, using GWAS-derived SNP markers (Qin et al., 2019; Ravelombola et al., 2019, 2020, 2021). Therefore, an approach combining MAS and GS through GEBVs, using associated SNP markers (Spindel et al., 2016; Zhang J. P. et al., 2016; Qin et al., 2019; Ravelombola et al., 2019, 2020, 2021; Ali et al., 2020) will be a good choice to do molecular breeding for SCN resistance in common beans and, also, for other quantitative traits in other plant species.

In addition, GA is affected by the trait self, such as heritability. The GH has been estimated and reported in animals and plants such as heifers (Nawaz et al., 2018), soybean (Xavier and Rainey, 2020), and safflower (Zhao et al., 2021). de los Campos et al. (2015) developed whole-genome regression methods to estimate the GH, which was defined as the proportion of variance of a trait that can be explained (in the population) by linear regression on a set of markers. In this study, the GH was also estimated by the rrBLUP model for resistance to the three SCN HG Types, 0, 2.5.7, and 1.2.3.5.6.7 (Supplementary Table 13, Supplementary Figure 14), as we did for GP estimations. The results indicated that the higher GH, the higher GP, similar as reported by Xavier and Rainey (2020) for yield and related traits in soybeans.

### Utility of Common Bean Resistance Accessions

From this study, 15 out of 315 (4.8%) common bean accessions were resistant to SCN, with FI ranging from 4.8 to 10; 62 (19.7%) accessions were moderately resistant (10 < FI < 30) for HG Type 0 (race 6). The 15 resistant accessions were PI343950, PI313630, PI313328, PI201329, PI201354, PI313445, PI313440, PI313444, PI319684, PI417616, PI313501, PI325614, PI430206, PI313733, and PI269209, which will be preferred sources for resistance to HG Type 0 (race 6).

To select common bean accessions with resistance to multiple SCN HG Types, we combined the data of the SCN resistance to HG Types, 2.5.7 and 1.2.3.5.6.7 from the Wen et al. (2019) report and the data. We then selected 37 accessions, having broad resistance with FI < 20 to both HG Types, 0 and 1.2.3.5.6.7, and FI < 30 to HG Type 2.5.7 (Table 1). The genetic diversity of the 37 accessions showed similar to the genetic organization of the entire 315 accession collections (Figure 8, Table 1). Most of the resistant accessions belonged to the ecogeographic race Durango of the Middle American gene pool, although other gene pools or races also contained SCN resistance. The accessions with the highest resistance to multiple HG Types (with FI < 12 to the three HG Types) were PI201329, PI201354, PI313445, PI325642 (all race Durango), PI313733 (Andean admixed), and PI417616 (admixed) (Table 1, Supplementary Table 1).

These resistant accessions can be used in common bean breeding programs as parents to develop new cultivars with resistance to multiple SCN HG Types. In this study, we observed that the SCN resistance commonly existed in common bean accessions. There were 15 out of 315 (4.8%) common bean accessions resistant to HG Type 0 (race 6) with FI < 10 (Supplementary Table 1). Based on the report by Wen et al. (2019), 19 out of 363 accessions (5.2%) were resistant to HG Type 2.5.7, and 160 out of 363 (44.1%) resistant to HG Type 1.2.3.5.6.7 with FI < 10.

Interestingly, there were much more common bean lines resistant to HG Type 1.2.3.5.6.7 than HG Type 2.5.7 and HG Type 0. This contrasts to the SCN resistance in soybean, which has fewer lines resistant to HG 1.2.3.5.6.7 as compared with HG Type 2.5.7, and much fewer lines as compared with HG Type 0 because a population of HG Type 1.2.3.5.6.7 generally has broader virulence than a population of HG Type 2.5.7 or HG Type 0. Although the FI on the HG Type indicator lines of the two SCN populations used by Wen et al. (2019) was not reported, it is possible that the mechanisms of SCN resistance differed between soybeans and common beans. If this is true, the different and broad-spectrum SCN resistance in common beans potentially provides excellent sources of SCN resistance to soybeans. SCN has been the most damaging pest in soybeans. Only a few sources available for resistance to multiple HG Types, particularly for resistance to HG Type 2.5.7 and 1.2.3.5.6.7, but none of them has been successfully deployed in commercial soybean cultivars. After the discovery of the SCN resistance genes in common beans, it will be possible to transfer the genes from common beans to soybeans through a transgenic approach.

## Conclusion

In this study, 15 accessions of the USDA common bean core collection were observed for the resistance to SCN HG Type 0 with FI at 4.8 to 9.4; six SNP markers, located on chromosomes Pv 04, 06, 07, 07, 09, and 11, respectively, were significantly associated with the resistance to this SCN HG Type 0. GWAS was also conducted for resistance to HG Type 2.5.7 and HG Type 1.2.3.5.6.7 based on published phenotypic data and the genotypic data from the BARCBean6K_3 chip. Eleven SNPs were associated with HG Type 2.5.7 resistance on chromosomes Pv01, 02, 03, 07, 09, and 11, and four SNPs with HG Type 1.2.3.5.6.7 resistance on chromosomes Pv 03, 06, 10, and 11. A gene model of the disease resistance family protein/LRR protein family, Phvul.004G104700, was close to the SNP marker ss715640464 at a distance of 8.98 Kbp in the same LD region of chromosome Pv04, suggesting that Phvul.004G104700 may be a candidate gene for the HG Type 0 resistance. GP was performed for resistance to three HG Types, using eight GP models (rrBLUP, gBLUP, Bayes A, Bayes B, BL, BRR, RF, and SVM), with BL showing the most promising results in terms of PA. The results showed that 400 SNPs or more had similar GS efficiency for resistance to either HG Type 0, 2.5.7, or 1.2.3.5.6.7, and the set of 20 significant SNP markers had the highest PA for GP. The six sets of different testing set sizes from 5 to 50% had similar r-values. Except for gBLUP (lower PA), all other seven models had similar PA. The averaged r-values were 0.40 for HG Type 0 resistance, 0.34 for HG Type 2.5.7, and 0.32 for HG Type 1.2.3.5.6.7. This study provides basic information for breeders to develop SCN-resistant common bean cultivars, using the USDA core germplasm accessions through MAS and GS in common beans.

## Data Availability Statement

The datasets presented in this study can be found in online repositories. The names of the repository/repositories and accession number(s) can be found in the article/Supplementary Material.

## Author Contributions

SC was the principal investigator (PI), performed phenotypic data collection and analysis, and revised and wrote part of the manuscript. TM and AS were the Co-PI of the project. QS developed the SNP Chip and genotyped the accessions. PG performed genotypic data collection, using the SNP Chip. AS performed genomic and statistical analysis and wrote the draft of the manuscript. HX assisted in the data analysis. All the authors have edited, reviewed, and approved the manuscript.

## Conflict of Interest

The authors declare that the research was conducted in the absence of any commercial or financial relationships that could be construed as a potential conflict of interest. The reviewer DZ declared a shared affiliation, with no collaboration, with one of the authors QS to the handling editor at the time of the review.
