# Supplementary figures and images for: Genome-Wide Association Study and Genomic Prediction for Soybean Cyst Nematode Resistance in USDA Common Bean (Phaseolus vulgaris) Core Collection
Source: Front Plant Sci. 2021 Jun 7;12:624156. doi: 10.3389/fpls.2021.624156 (PMC8215670; doi:10.3389/fpls.2021.624156)

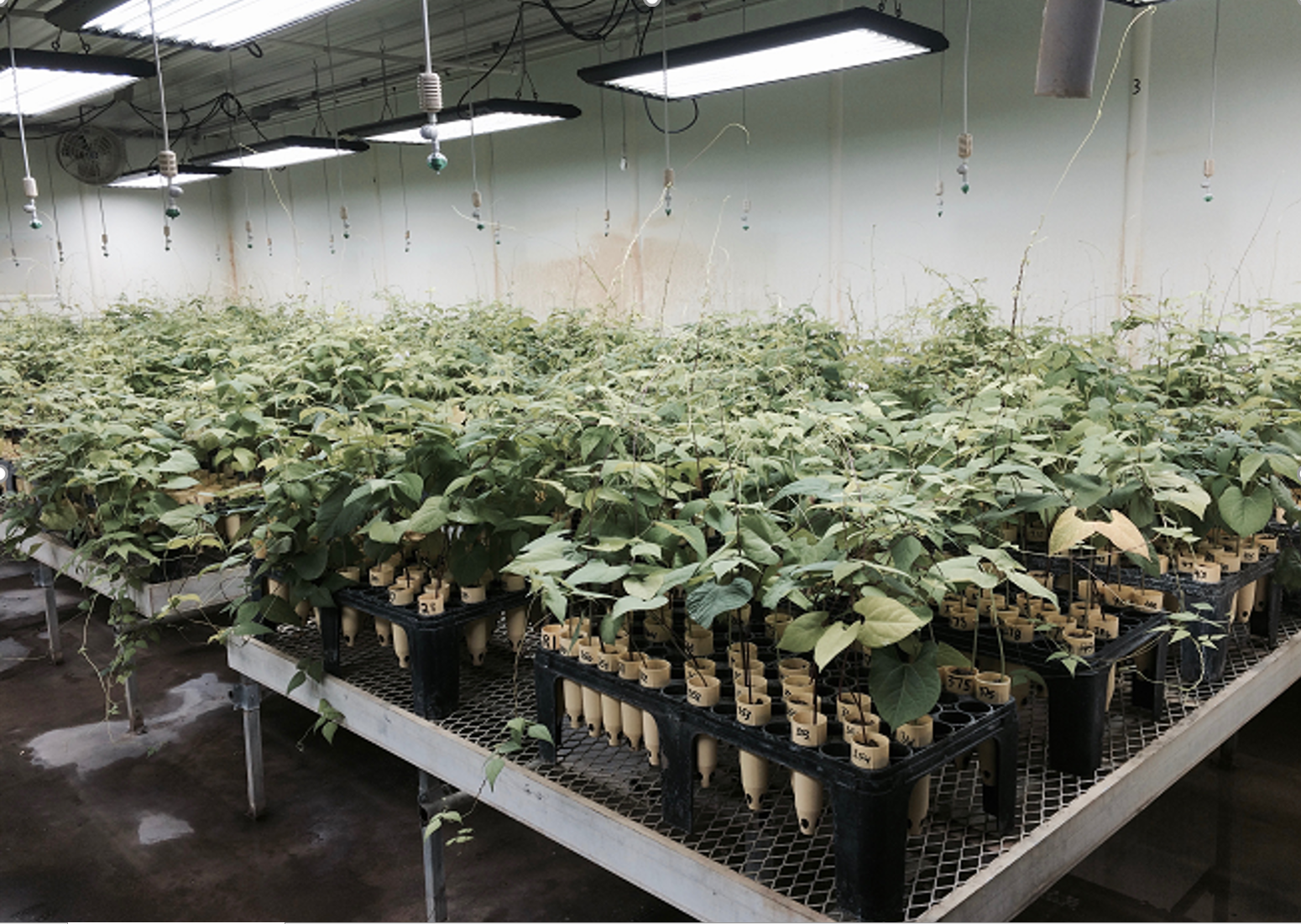

Supplement: Supplementary file 1 [file Data_Sheet_1.zip › Supplementary Figures 1-14_images/Image 1.TIF]

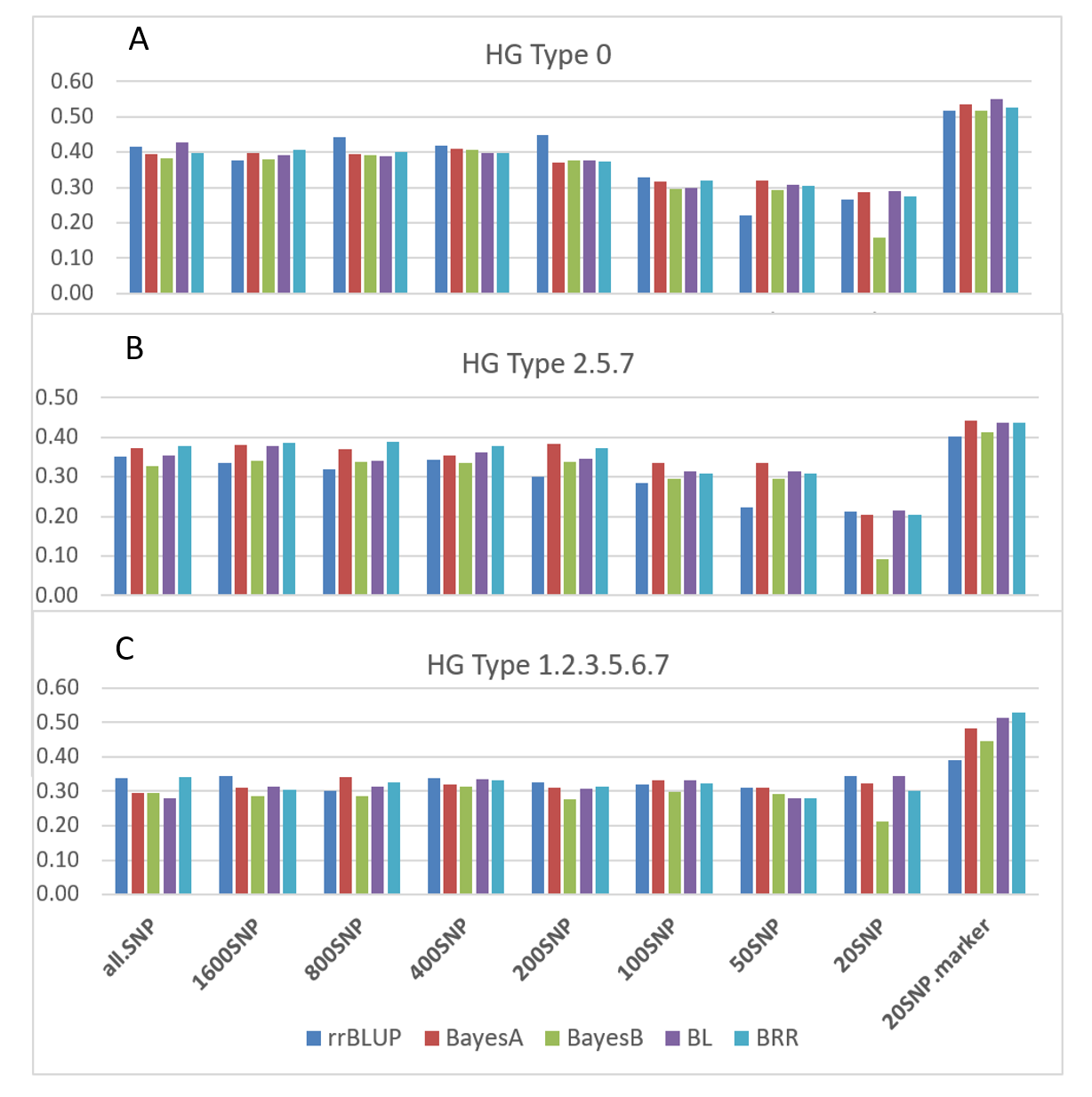

Supplement: Supplementary file 1 [file Data_Sheet_1.zip › Supplementary Figures 1-14_images/Image 10.TIF]

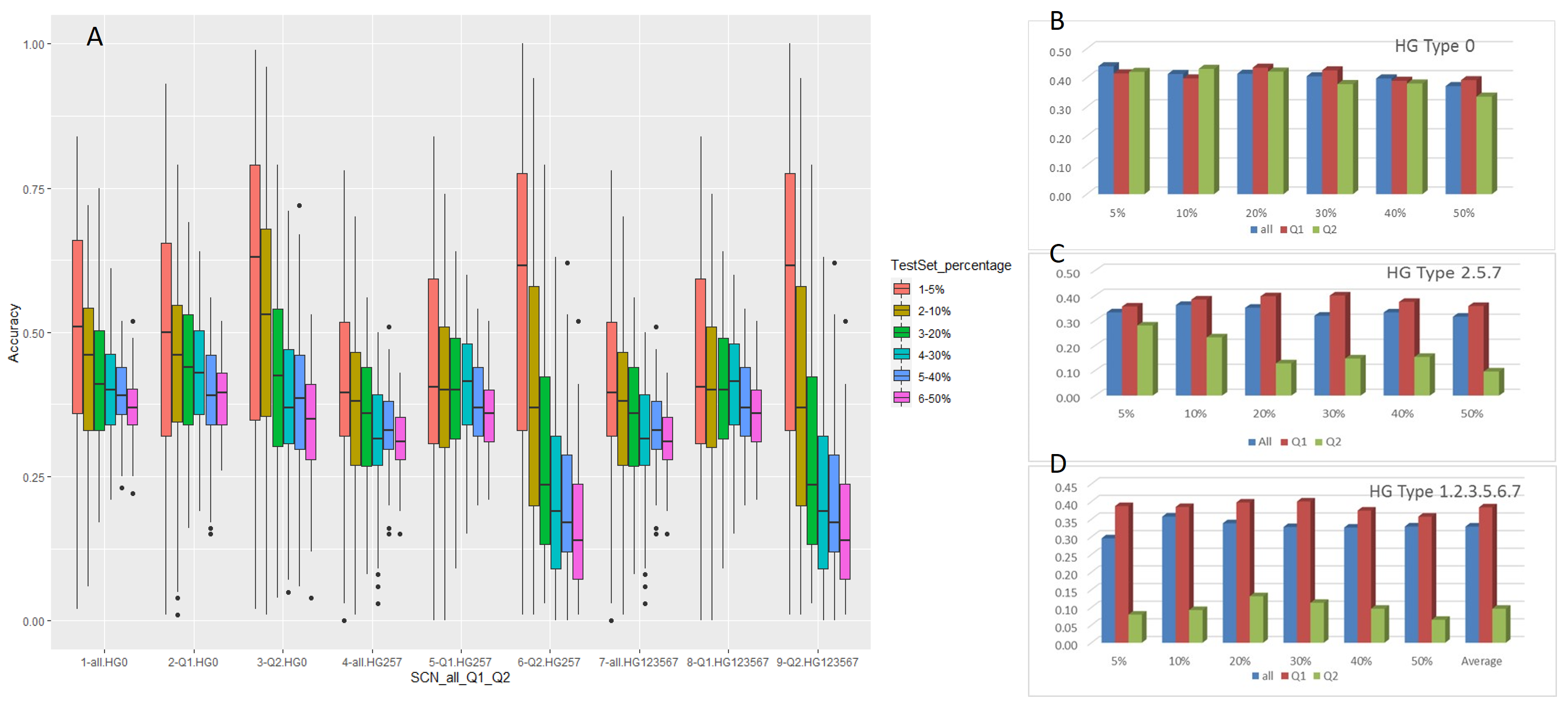

Supplement: Supplementary file 1 [file Data_Sheet_1.zip › Supplementary Figures 1-14_images/Image 11.TIF]

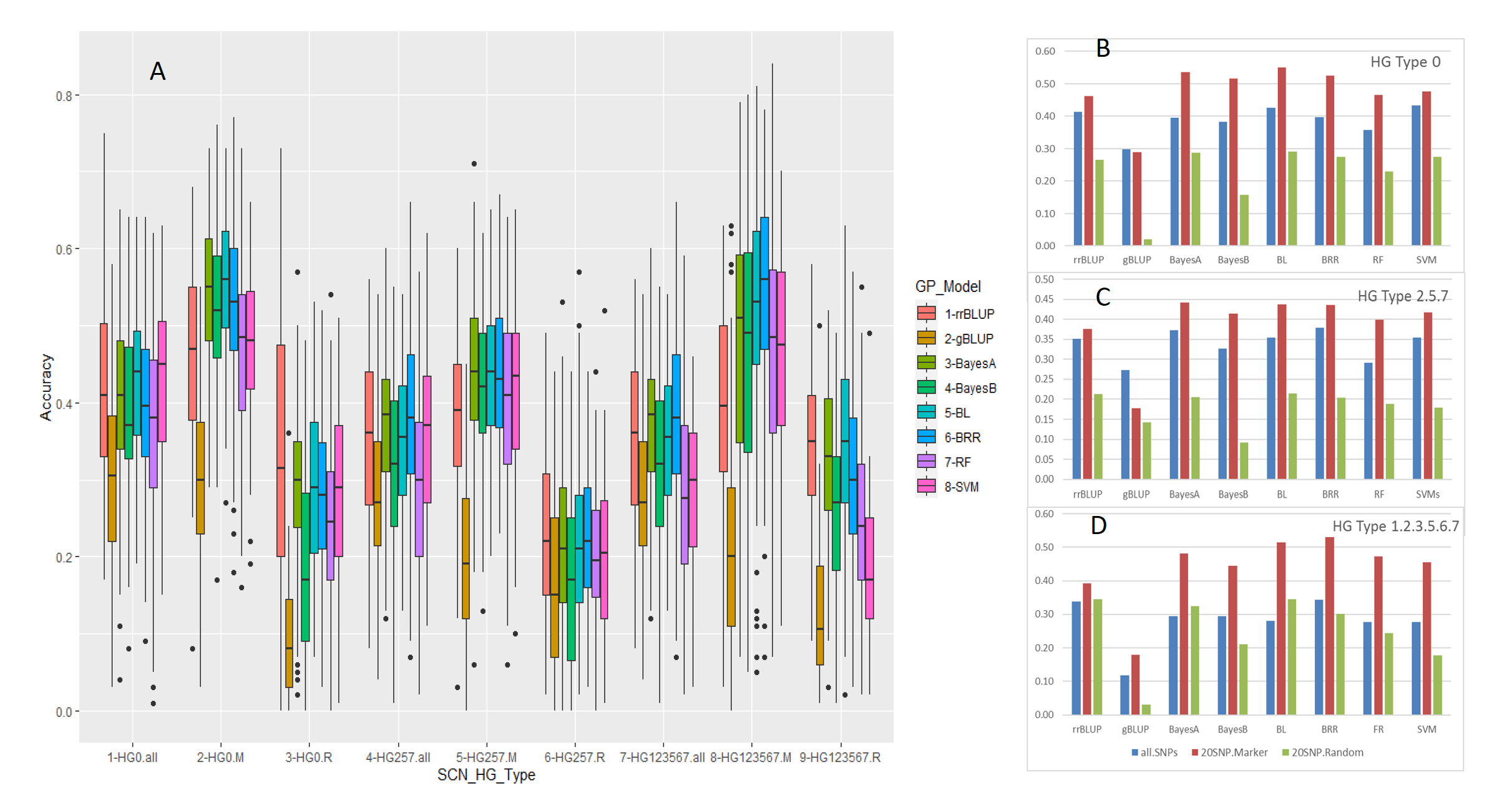

Supplement: Supplementary file 1 [file Data_Sheet_1.zip › Supplementary Figures 1-14_images/Image 12.TIF]

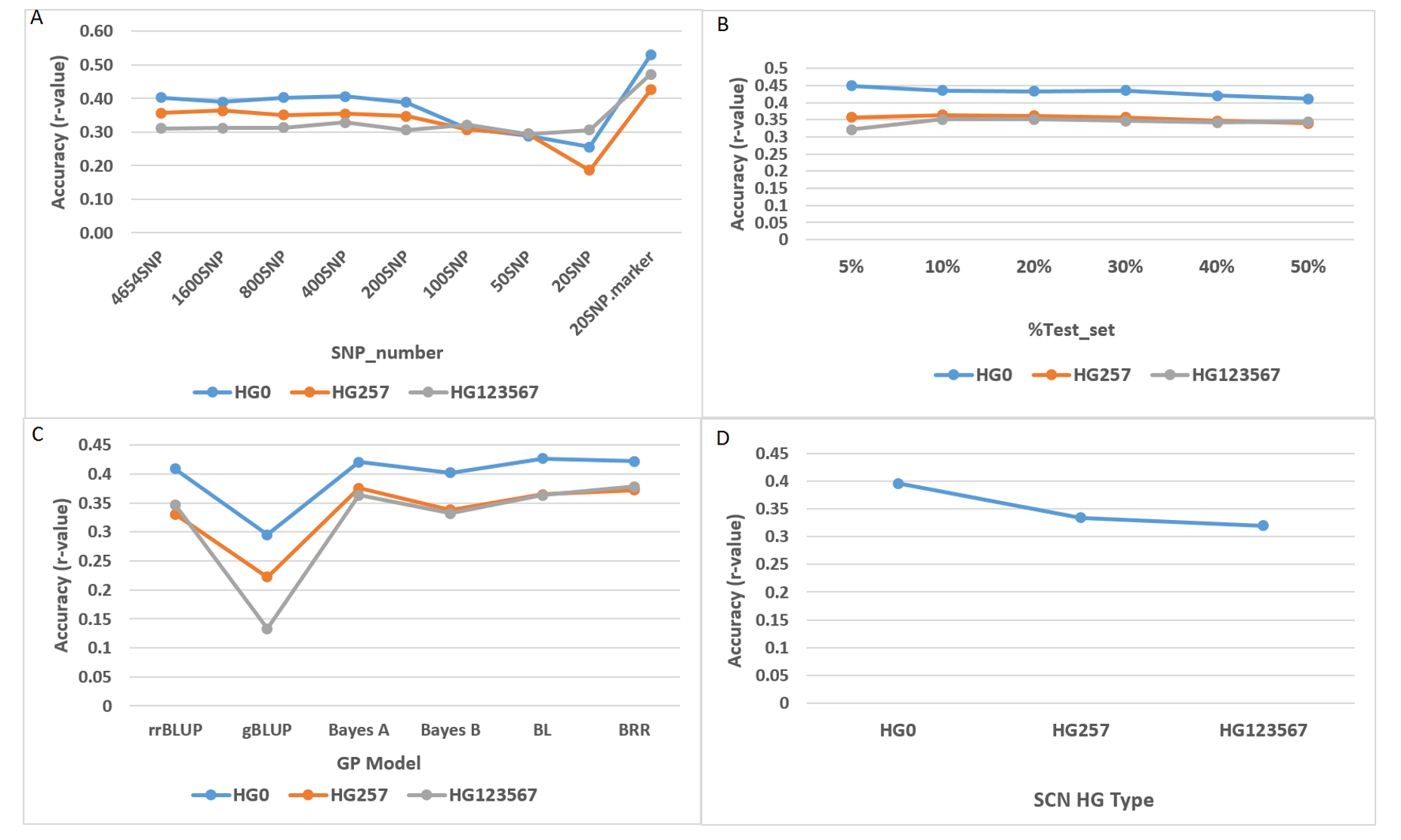

Supplement: Supplementary file 1 [file Data_Sheet_1.zip › Supplementary Figures 1-14_images/Image 13.TIF]

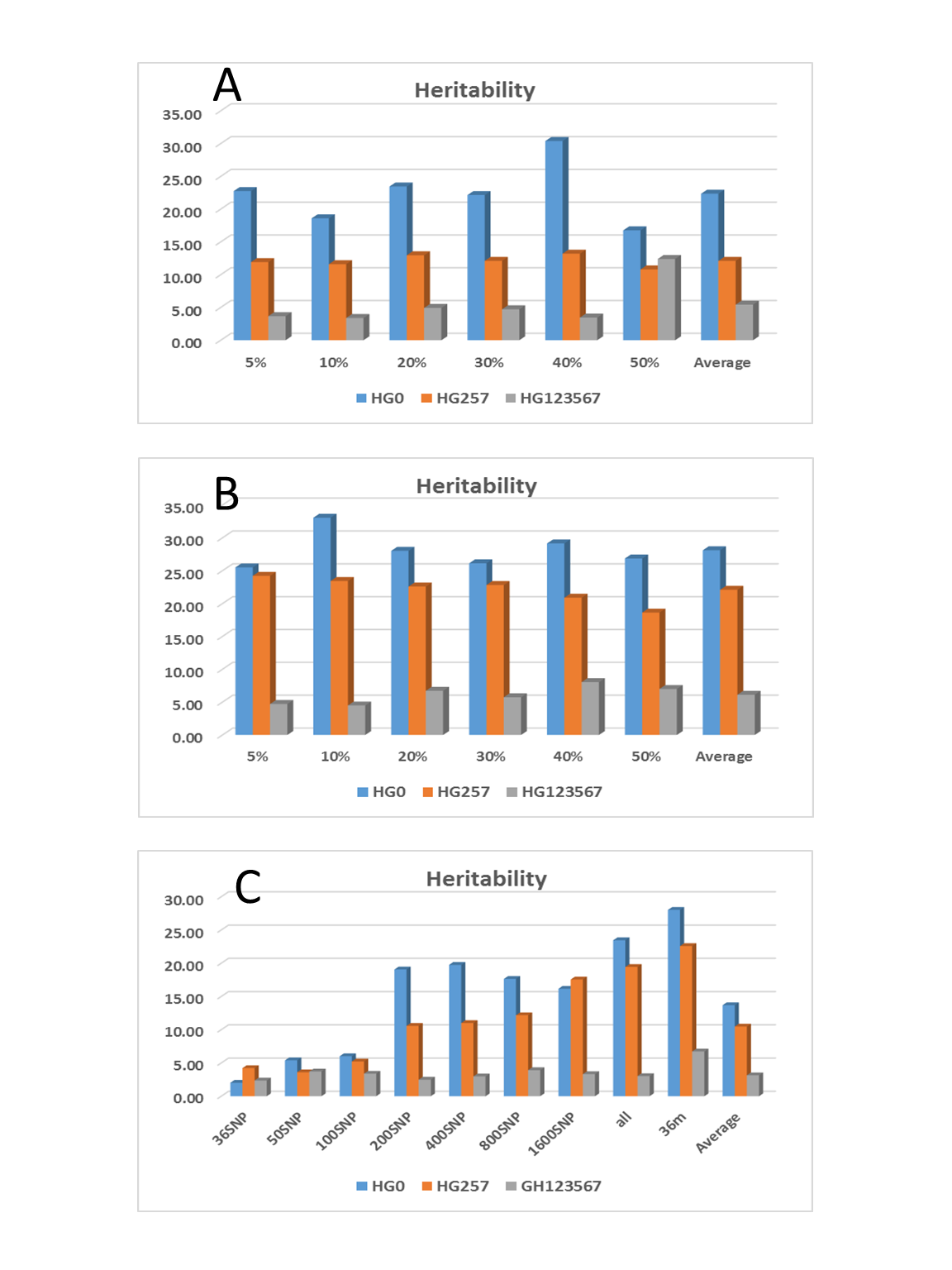

Supplement: Supplementary file 1 [file Data_Sheet_1.zip › Supplementary Figures 1-14_images/Image 14.TIF]

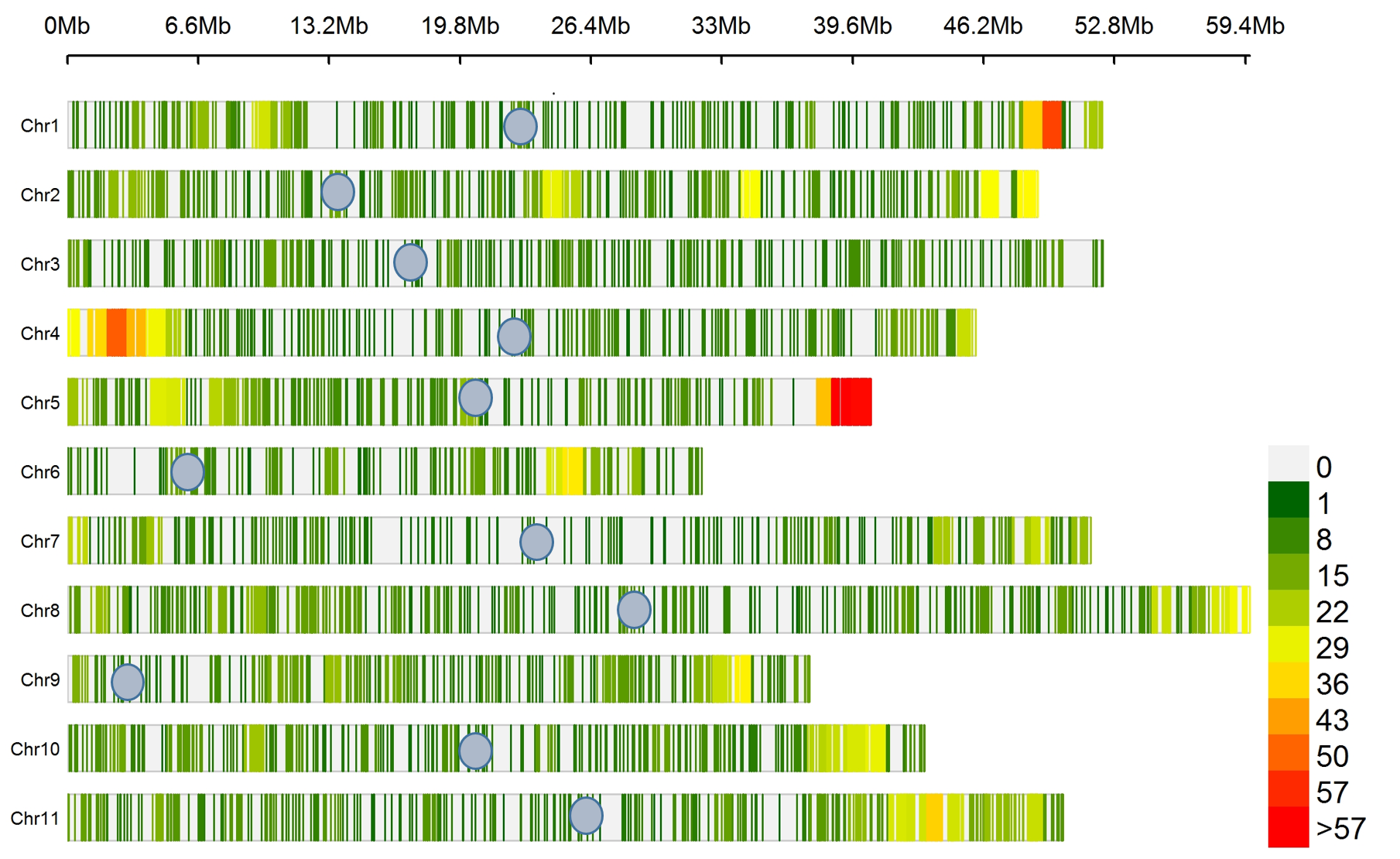

Supplement: Supplementary file 1 [file Data_Sheet_1.zip › Supplementary Figures 1-14_images/Image 2.TIF]

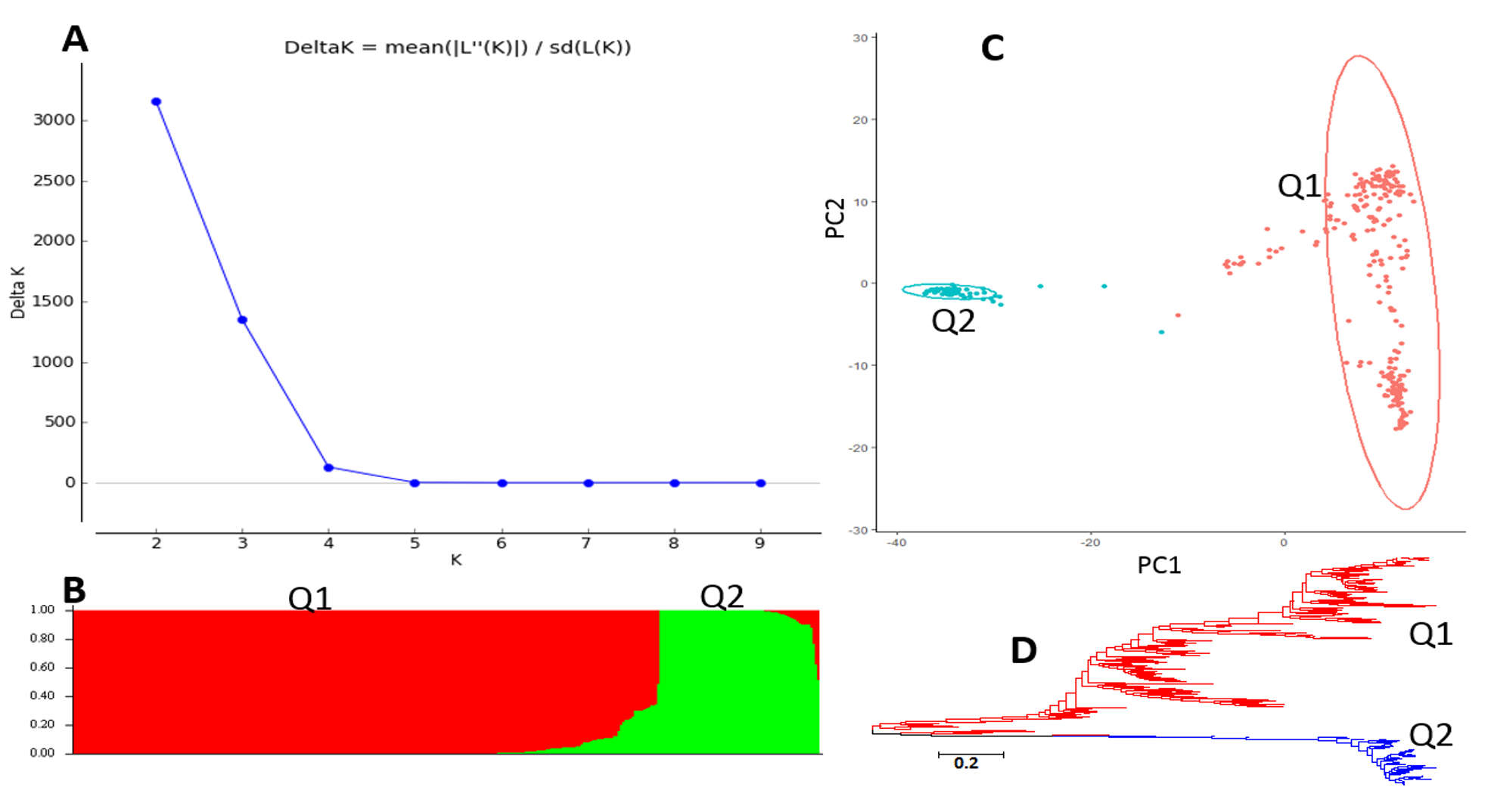

Supplement: Supplementary file 1 [file Data_Sheet_1.zip › Supplementary Figures 1-14_images/Image 3.TIF]

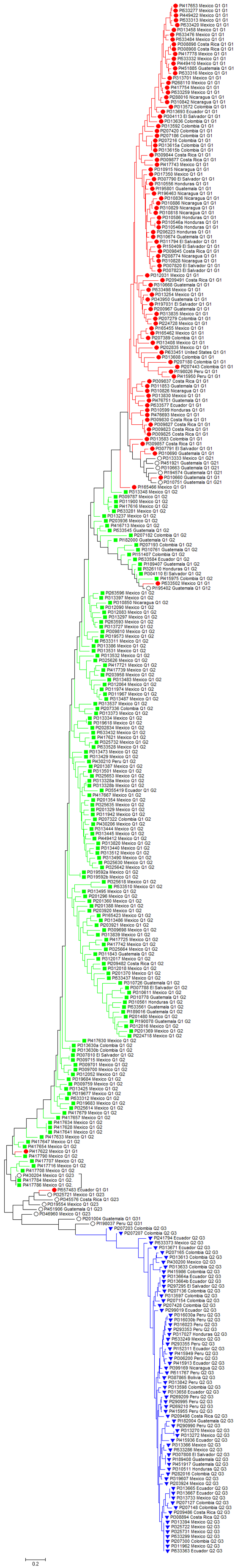

Supplement: Supplementary file 1 [file Data_Sheet_1.zip › Supplementary Figures 1-14_images/Image 4.TIF]

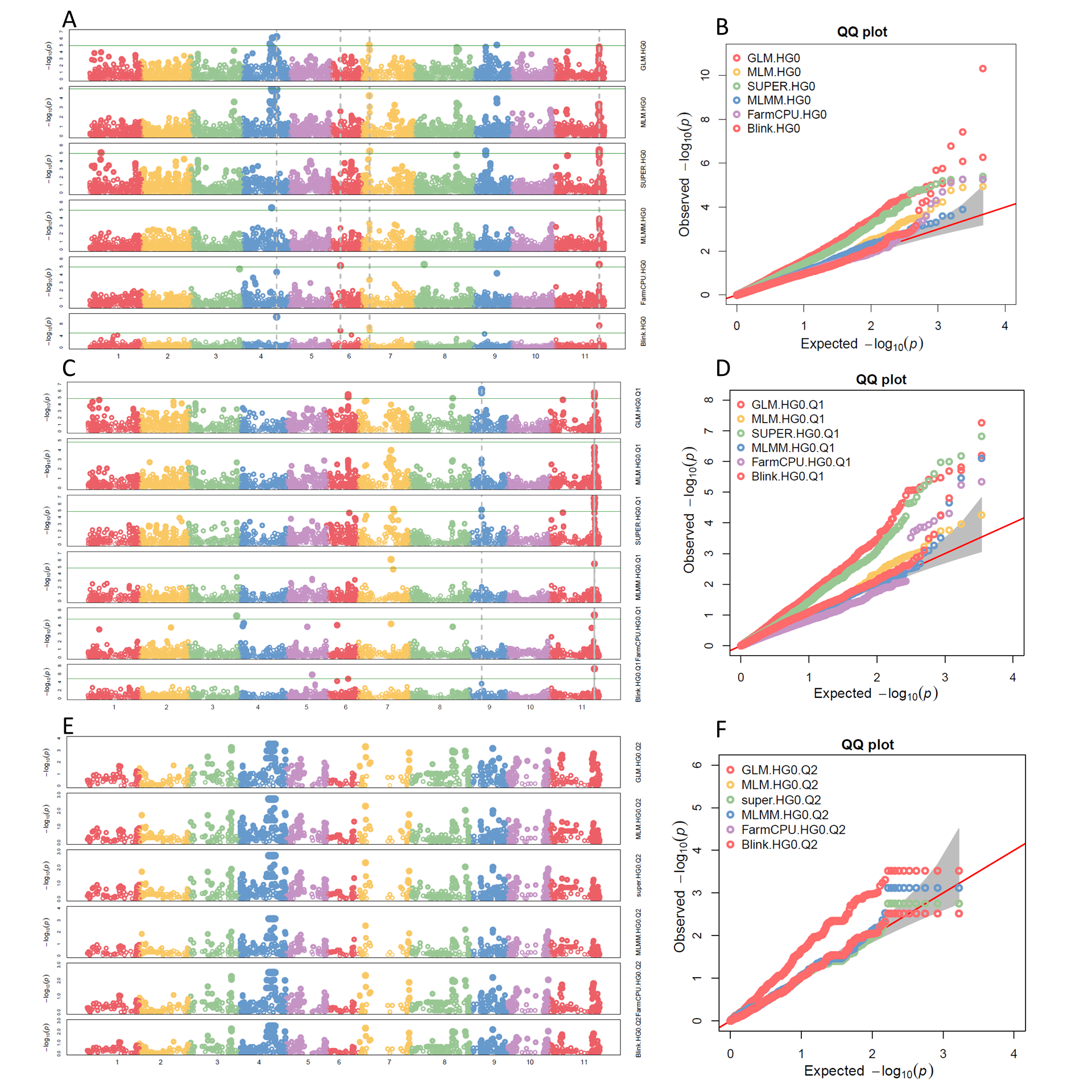

Supplement: Supplementary file 1 [file Data_Sheet_1.zip › Supplementary Figures 1-14_images/Image 5.TIF]

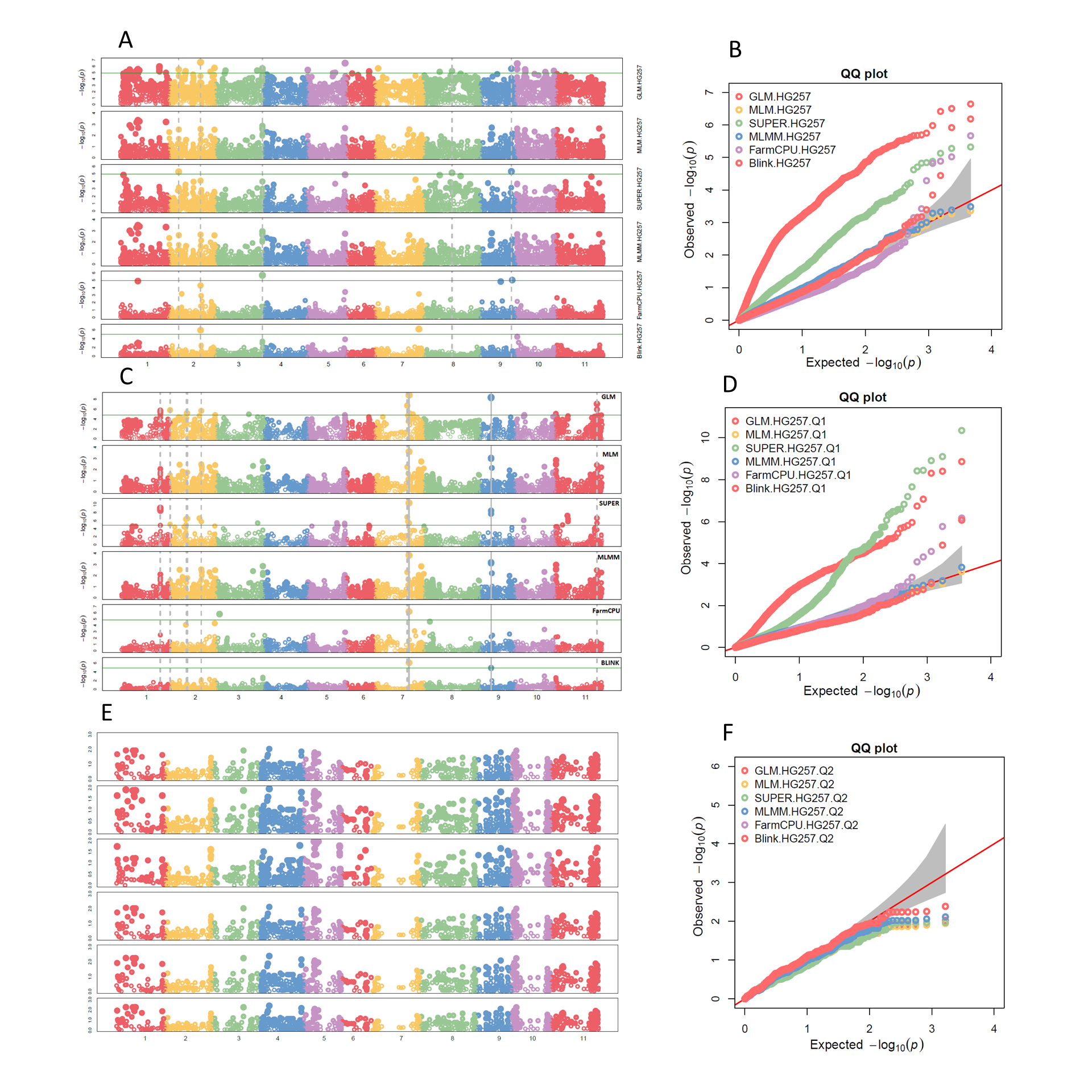

Supplement: Supplementary file 1 [file Data_Sheet_1.zip › Supplementary Figures 1-14_images/Image 6.TIF]

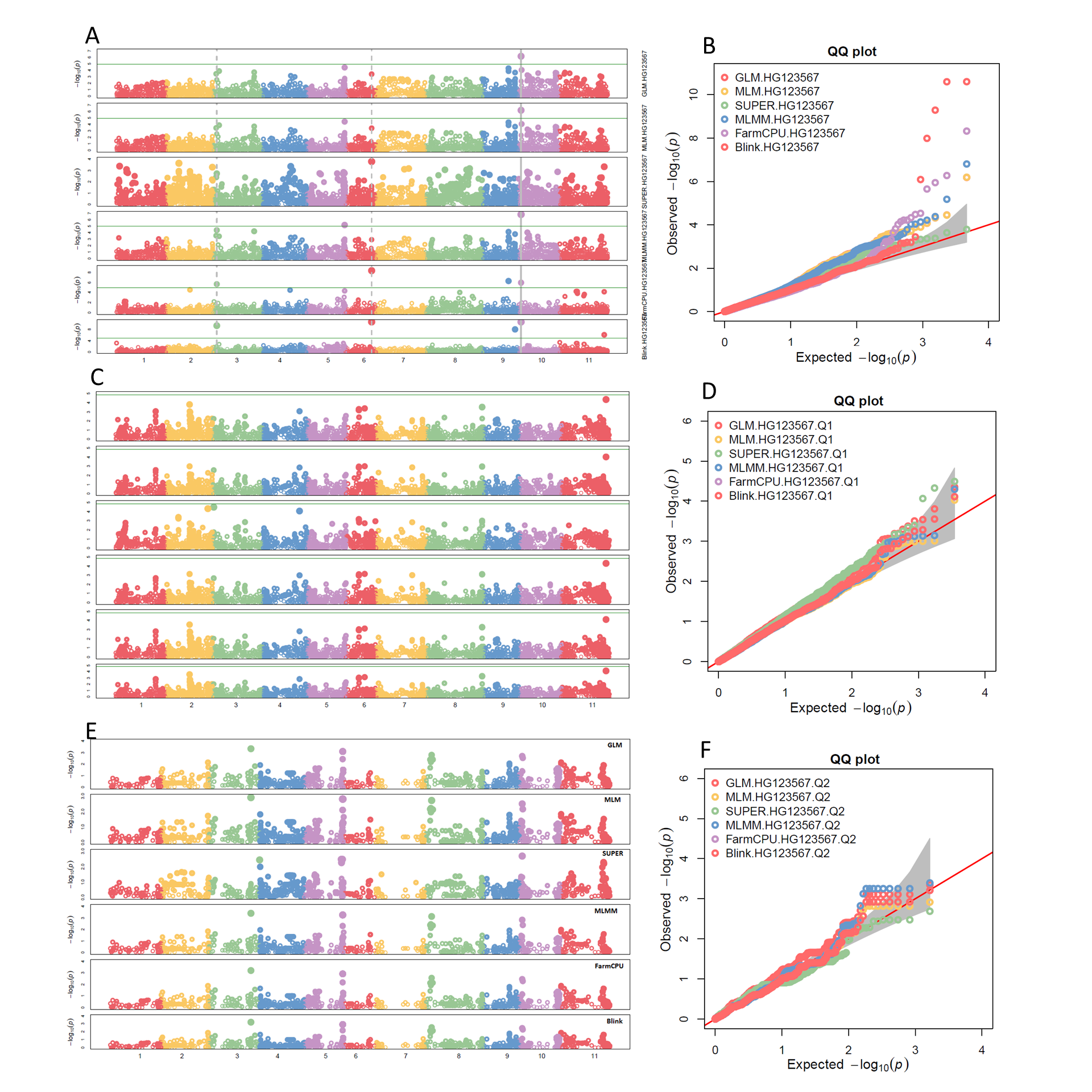

Supplement: Supplementary file 1 [file Data_Sheet_1.zip › Supplementary Figures 1-14_images/Image 7.TIF]

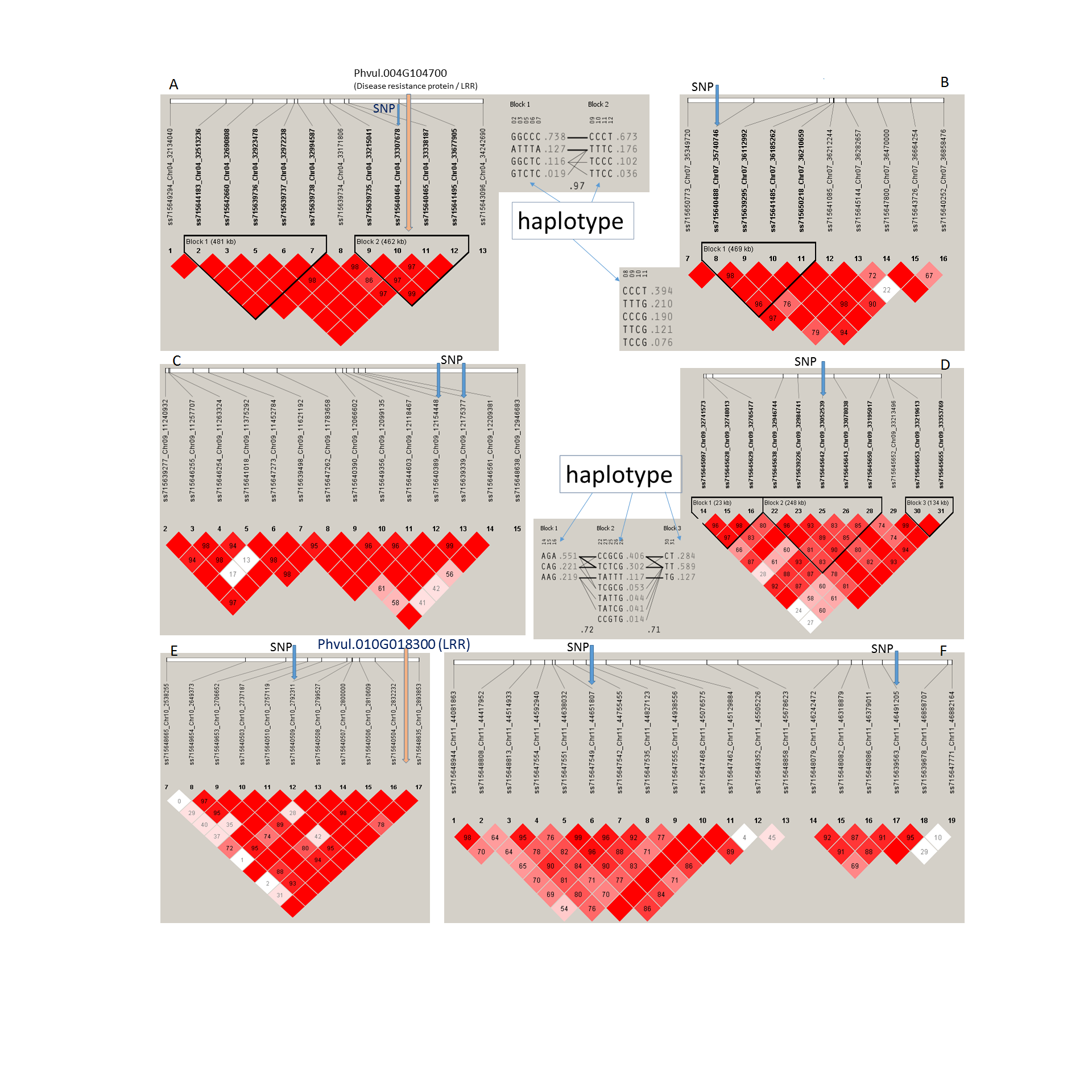

Supplement: Supplementary file 1 [file Data_Sheet_1.zip › Supplementary Figures 1-14_images/Image 8.TIF]

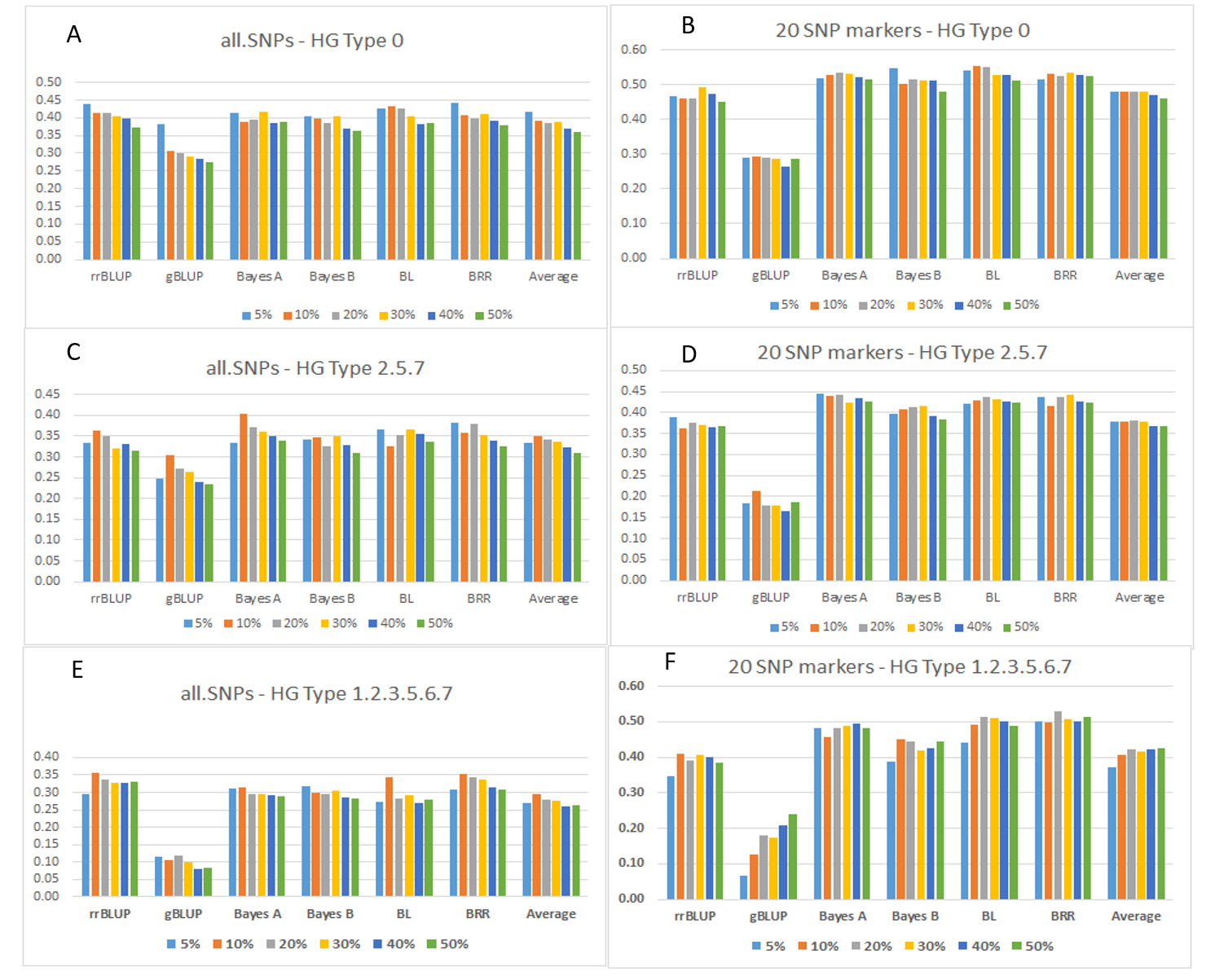

Supplement: Supplementary file 1 [file Data_Sheet_1.zip › Supplementary Figures 1-14_images/Image 9.TIF]
